# Neutrophils fuel effective immune responses through gluconeogenesis and glycogenesis

**DOI:** 10.1016/j.cmet.2021.03.018

**Published:** 2021-05-04

**Authors:** Pranvera Sadiku, Joseph A. Willson, Eilise M. Ryan, David Sammut, Patricia Coelho, Emily R. Watts, Robert Grecian, Jason M. Young, Martin Bewley, Simone Arienti, Ananda S. Mirchandani, Manuel A. Sanchez Garcia, Tyler Morrison, Ailing Zhang, Leila Reyes, Tobias Griessler, Privjyot Jheeta, Gordon G. Paterson, Christopher J. Graham, John P. Thomson, Kenneth Baillie, A.A. Roger Thompson, Jessie-May Morgan, Abel Acosta-Sanchez, Veronica M. Dardé, Jordi Duran, Joan J. Guinovart, Gio Rodriguez-Blanco, Alex Von Kriegsheim, Richard R. Meehan, Massimiliano Mazzone, David H. Dockrell, Bart Ghesquiere, Peter Carmeliet, Moira K.B. Whyte, Sarah R. Walmsley

## Main text

(*Cell Metabolism* 33, 411–423.e1–e4; February 2, 2021)

In the originally published version of this article, an earlier draft of Figure 5 was mistakenly included. This has now been replaced with the final version, which includes data generated during the revision process. Updated figure panels now include bacterial killing of *Staphylococcus aureus* (SH1000) (Figure 5B), baseline ATP levels (Figure 5D), glycolytic response to SH1000 (Figures 5E and 5F), and tracing of U-13C glutamine into F1,6BP (Figure 5R). Figure 5G has been removed and replaced by 5E; 5L has been removed and replaced by 5D. The remaining panels have been renumbered in line with the figure legend and Results text. The figure legend in the originally published article is correct and corresponds to the updated figure. This error does not affect the data and conclusions of the paper. The authors sincerely apologize for any confusion that this error may have caused.

**Figure:**
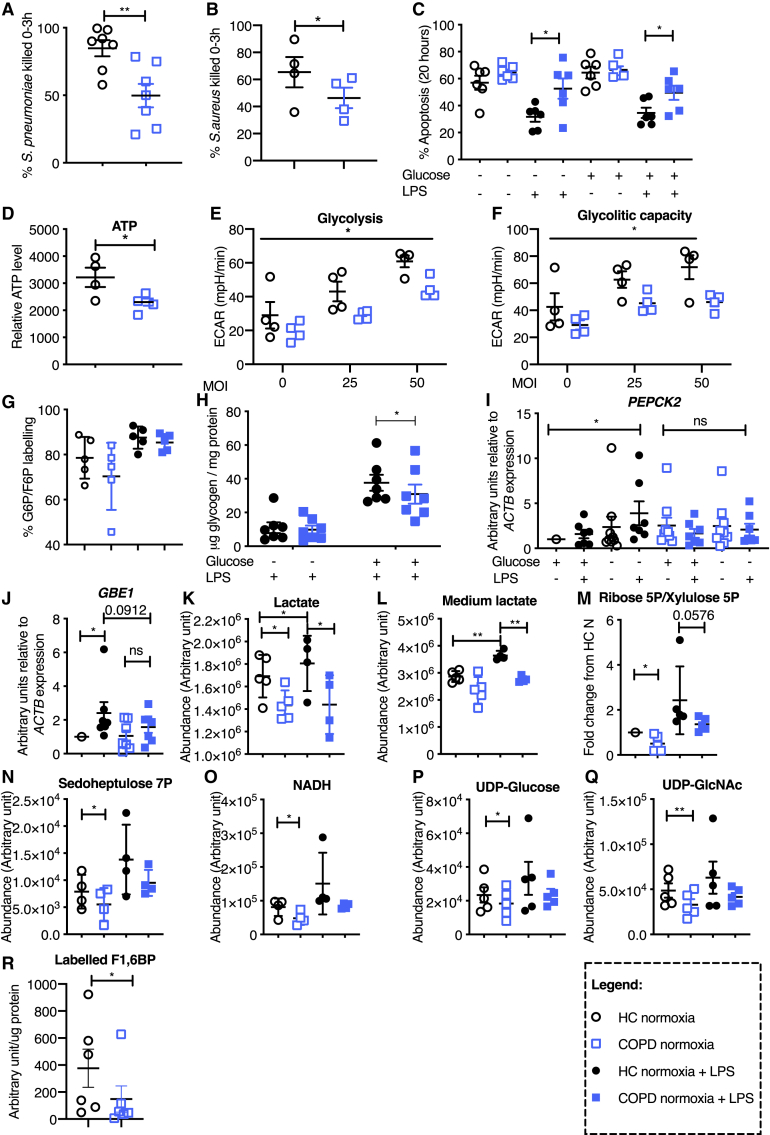
Figure 5. COPD Peripheral Blood Neutrophils Are Unable to Regulate Their Glycogen Synthesis, Resulting in Diminished Intracellular Glycogen Stores, Defective Bacterial Killing, and Survival (corrected)

**Figure:**
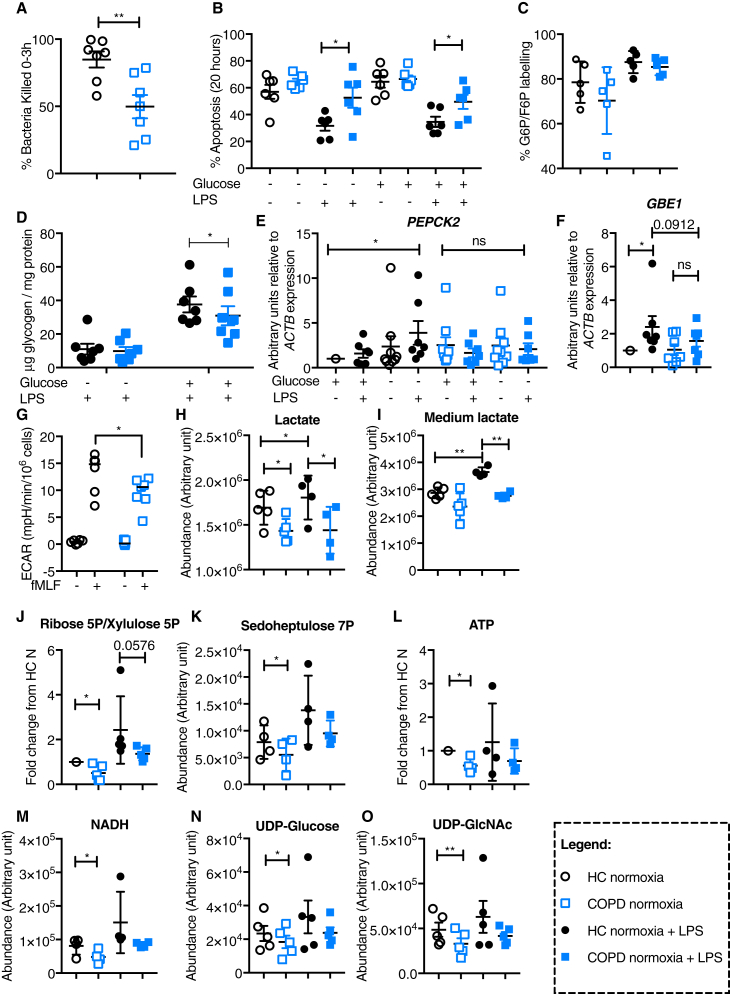
Figure 5. COPD Peripheral Blood Neutrophils Are Unable to Regulate Their Glycogen Synthesis, Resulting in Diminished Intracellular Glycogen Stores, Defective Bacterial Killing, and Survival (original)

