# Supplementary material for: Neutrophils Fuel Effective Immune Responses through Gluconeogenesis and Glycogenesis
Source: Cell Metab. 2021 Feb 2;33(2):411–423.e4. doi: 10.1016/j.cmet.2020.11.016 (PMC7863914; doi:10.1016/j.cmet.2020.11.016)
Supplement: Document S1. Figures S1–S6 and Table S1 [file mmc1.pdf]

Cell Metabolism, Volume *33*

Supplemental information

Neutrophils Fuel Effective Immune Responses through Gluconeogenesis and Glycogenesis

Pranvera Sadiku, Joseph A. Willson, Eilise M. Ryan, David Sammut, Patricia Coelho, Emily R. Watts, Robert Grecian, Jason M. Young, Martin Bewley, Simone Arienti, Ananda S. Mirchandani, Manuel A. Sanchez Garcia, Tyler Morrison, Ailing Zhang, Leila Reyes, Tobias Griessler, Privjyot Jheeta, Gordon G. Paterson, Christopher J. Graham, John P. Thomson, Kenneth Baillie, A.A. Roger Thompson, Jessie-May Morgan, Abel Acosta-Sanchez, Veronica M. Dardé, Jordi Duran, Joan J. Guinovart, Gio Rodriguez-Blanco, Alex Von Kriegsheim, Richard R. Meehan, Massimiliano Mazzone, David H. Dockrell, Bart Ghesquiere, Peter Carmeliet, Moira K.B. Whyte, and Sarah R. Walmsley

Supplemental Data

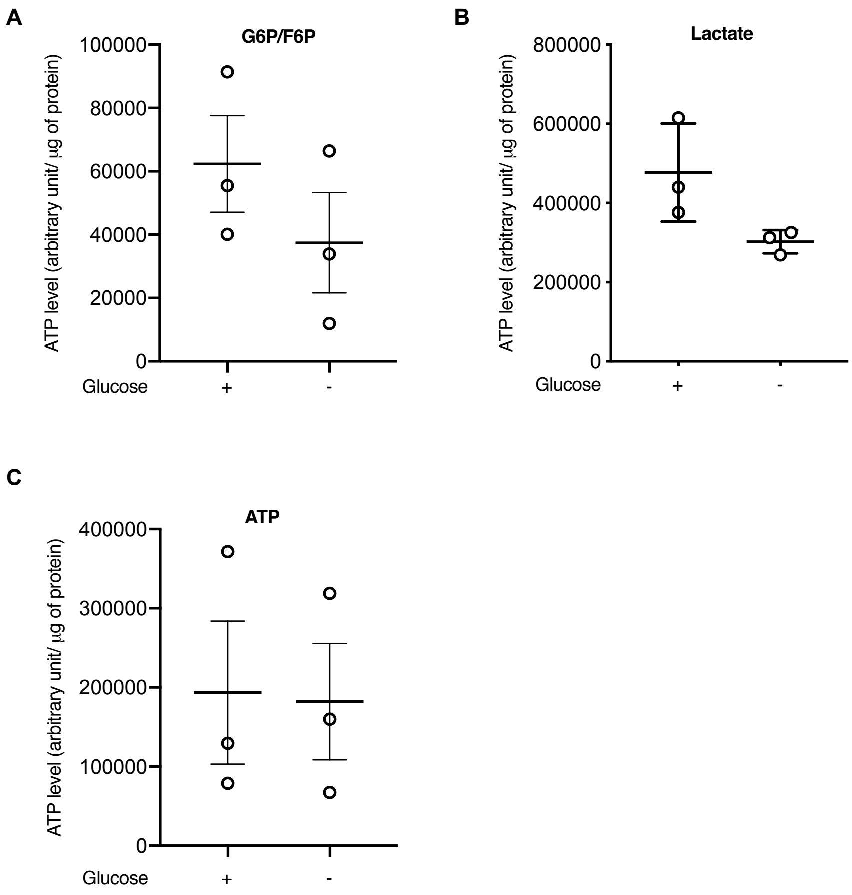

Figure S1. Total abundance of glucose-6-phosphate, lactate and ATP normalised to the protein content. Related to figure 2

Total abundance of glucose-6-phosphate (A), lactate (B) and ATP (C) normalised to the protein content are shown. Data represent mean ±SEM, n=3.

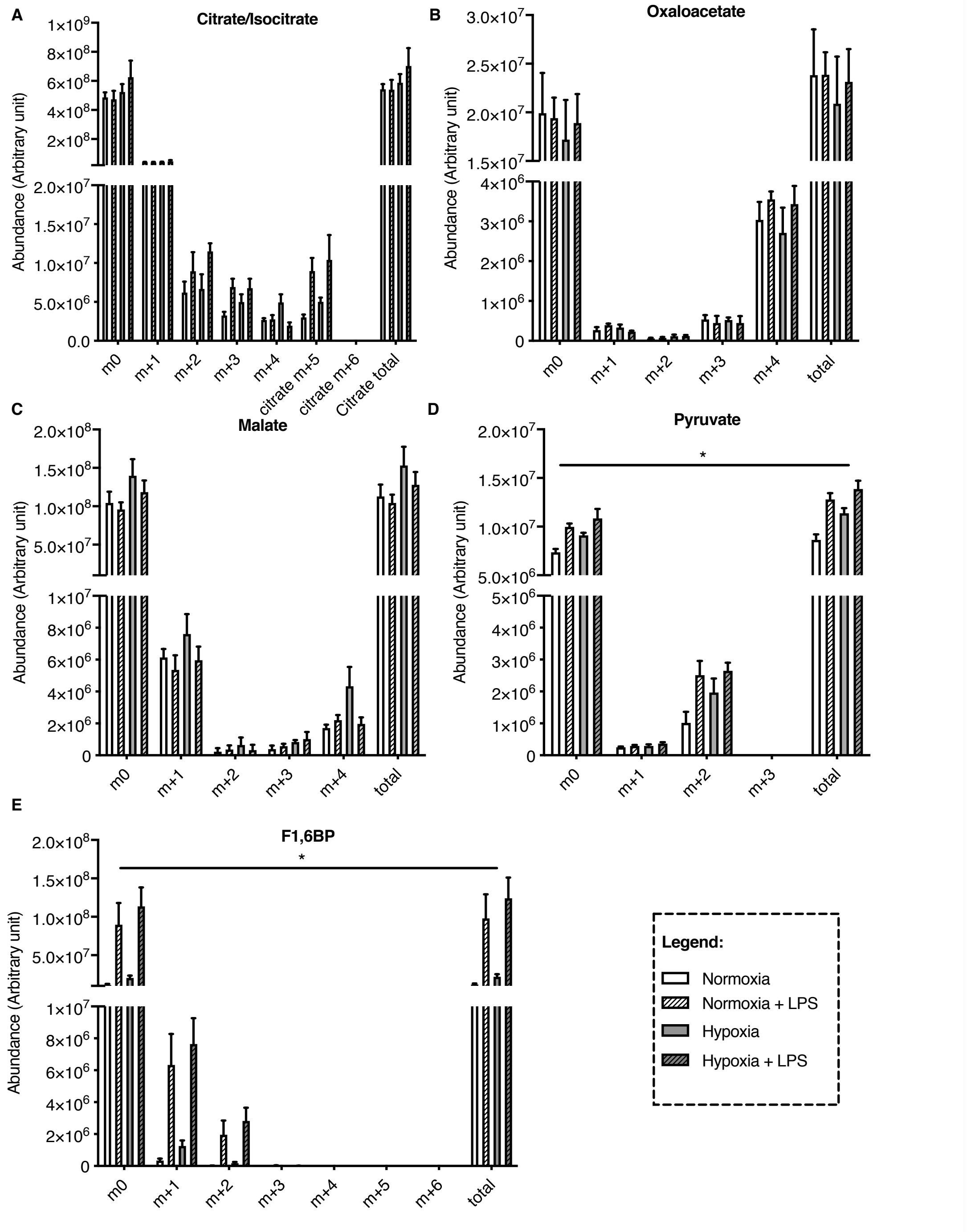

Figure S2. TCA intermediary isotopologue profiles following 4 hours of U-^{13}C glutamine culture. Related to figure 3

Neutrophils were cultured with U-^{13}C glutamine for 4 h in the presence and absence of LPS under normoxia and hypoxia. Full isotopologue profiles generated through U-^{13}C glutamine utilization are shown for citrate/isocitrate (A), oxaloacetate (B), malate (C), pyruvate (D) and fructose-1,6-bisphosphate (F1,6BP) (E). Data representative of mean ± SEM, n=4. Statistical significance was determined by 2-way ANOVA with Tukey's multiple comparisons test. *$P<0.05$, ****$P<0.0001$.

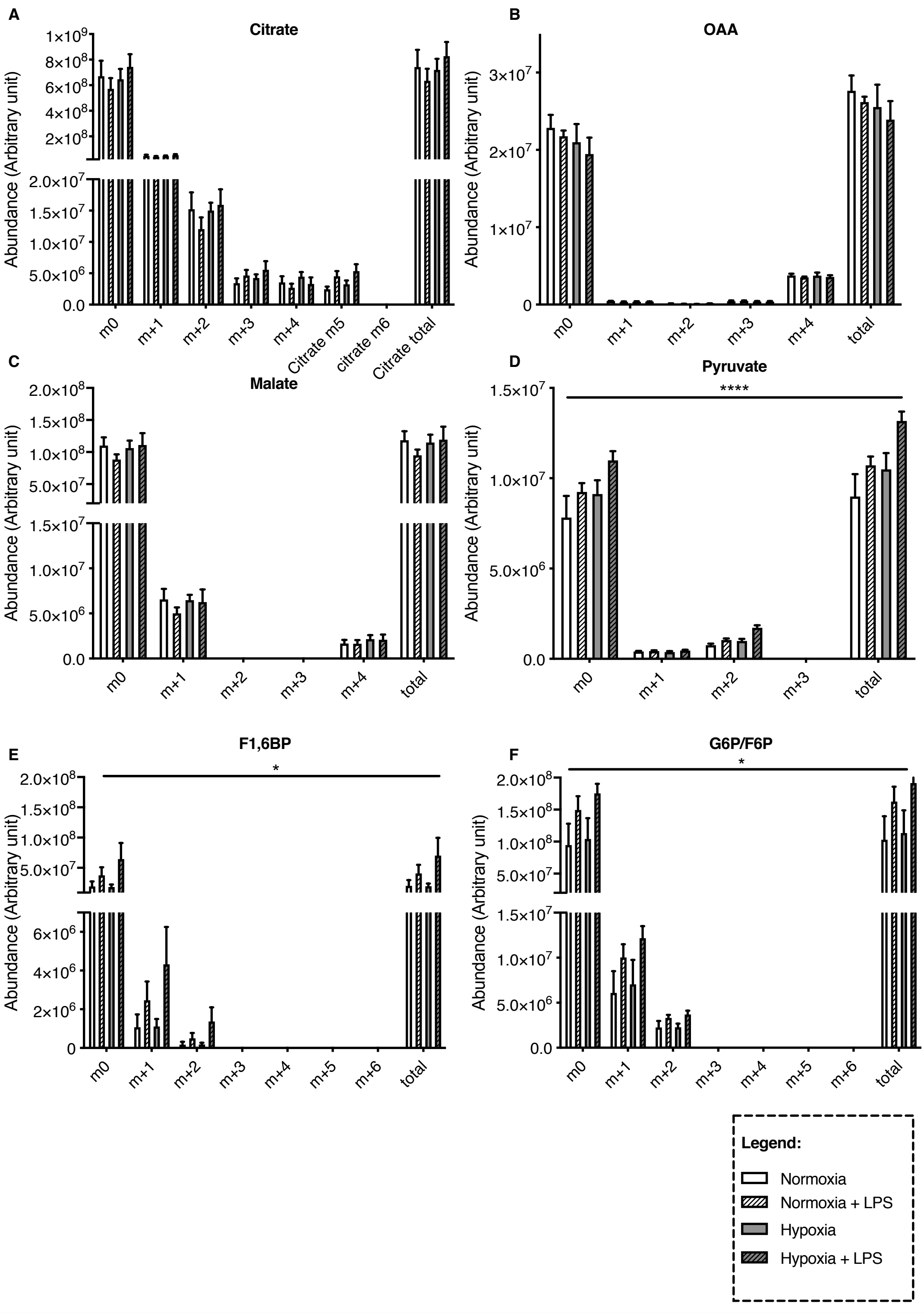

Figure S3. TCA intermediary isotopologue profiles following 2 hours of U-^{13}C glutamine culture. Related to figure 3

Neutrophils were cultured with U-^{13}C glutamine for 2 h in the presence and absence of LPS under normoxia and hypoxia. Full isotopologue profiles generated through U-^{13}C glutamine utilization are shown for citrate/isocitrate (A), oxaloacetate (B), malate (C), pyruvate (D), fructose-1,6-bisphosphate (F1,6BP) (E) and glucose-6-phosphate/fructose-6-phosphate (F). Data representative of mean ± SEM, n=4. Statistical significance was determined by 2-way ANOVA with Tukey's multiple comparisons test. *P<0.05, ****P<0.0001.

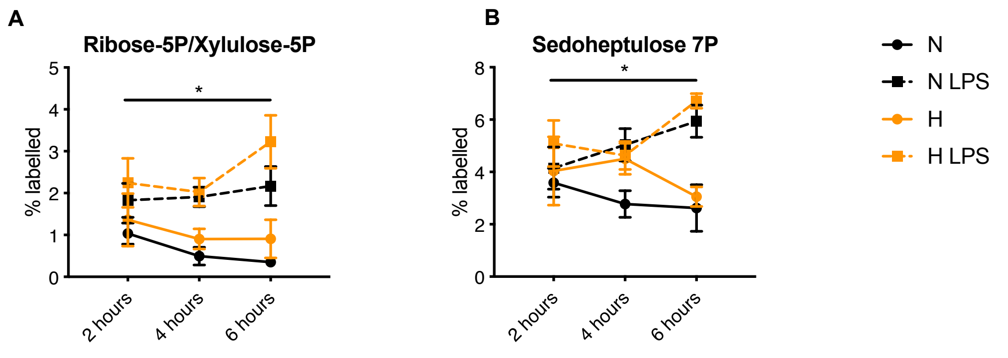

Figure S4. % labelling of PPP metabolites following 6 hours of U-^{13}C glutamine culture. Related to figure 3

Neutrophils were cultured in the presence of 13C-Glutamine for 2 (n=4), 4 (n=4) and 6 h (n=3). LC-MS time-course analyses of neutrophil % labelling of PPP metabolites Ribose-5 phosphate/xylulose-5 phosphate and Seduheptulose-7 phosphate, Data represent mean ±SEM. P values obtained via 2-way ANOVA.

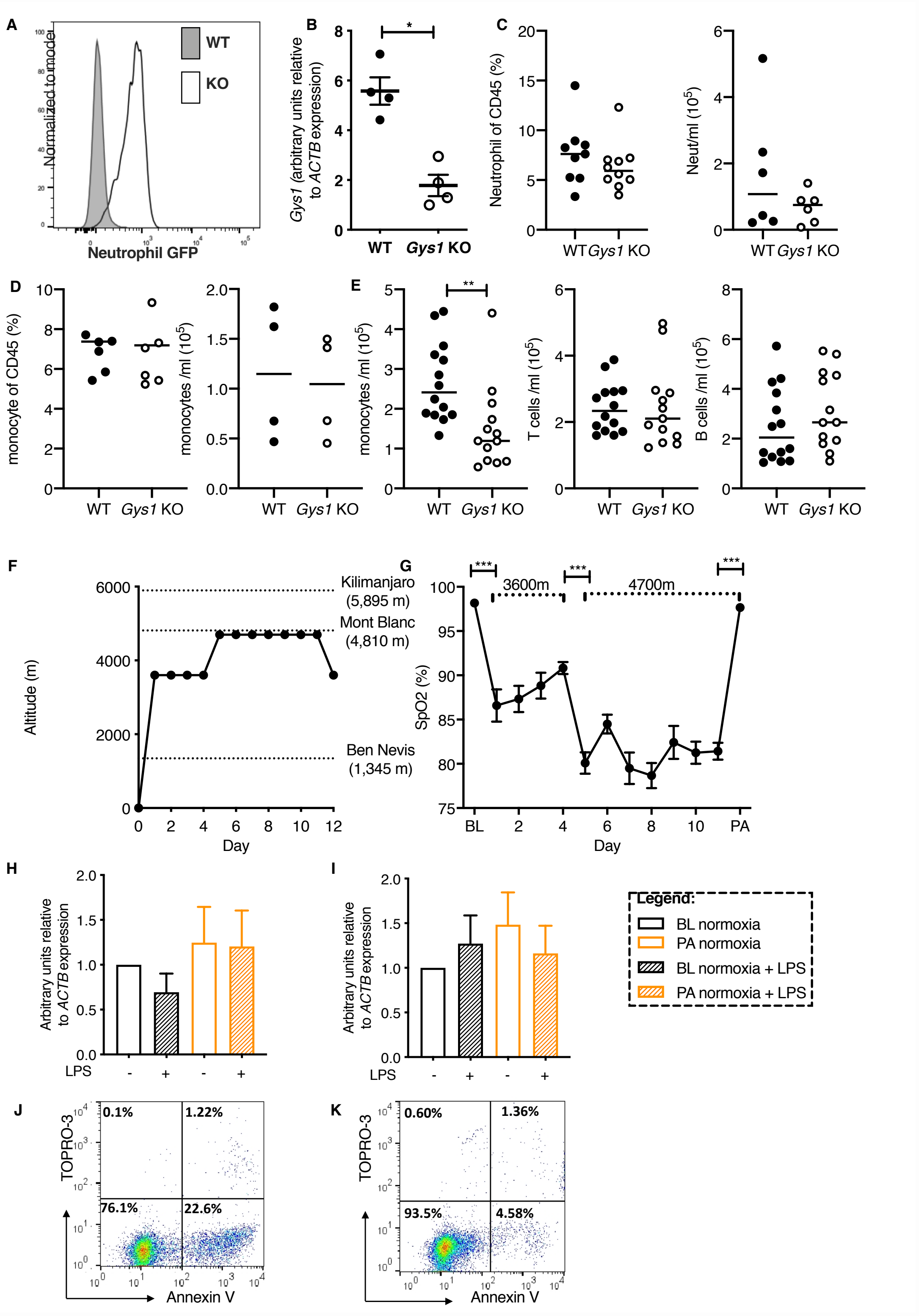

Figure S5. Gys1 deficiency and altitude induced systemic hypoxia regulate circulating blood leukocyte numbers and survival in mice and humans. Related to figure 4

Representative histogram of neutrophil expression of MRP8 driven GFP by flow cytometry comparing *Gys1* $^{lox/lox}$ MRP8-Cre$^{+/-}$ knockout (*GYS1* KO) mice, with *Gys1* $^{lox/lox}$ MRP8-Cre$^{-/-}$ wildtype (WT) (A). Quantitative PCR analysis of *Gys1* transcript expression in WT and Gys1 knockout mice (B), n=4. Proportion and number of circulating neutrophils (C) and monocytes (D) in WT and *GYS1*KO mice at baseline. Blood monocyte, T cell and B cell numbers in WT and KO mice 24 hours post *Staphylococcus aureus* infection (E). Graphical representation of subject exposure to high altitude following travel into La Paz, Bolivia (3,600m) and ascension to Huayna Potosí base camp (4,700m) (F). Mean peripheral blood oxygen saturation SpO2 was measured using pulse oximetry at baseline (BL), daily at high altitude, and three-months post-altitude (PA) (G), n = 12. Transcript expression of GBE1 (H), n=8 and GLUT1 (I), n=7 in peripheral blood neutrophils at BL and 3 months PA following 4 h of culture in the presence and absence of LPS under normoxia. Representative flow cytometry plots of Annexin V/TO-PRO-3 staining of BL (J) and 3 months PA (K) peripheral blood neutrophils following stimulation with LPS for 20 h under hypoxia. Data represent mean ± SEM. Statistical significance was determined by unpaired *t* test (B, E) and between two means using post hoc Holm-Sidak multiple comparison tests (G). **P<0.01, ***P<0.005.

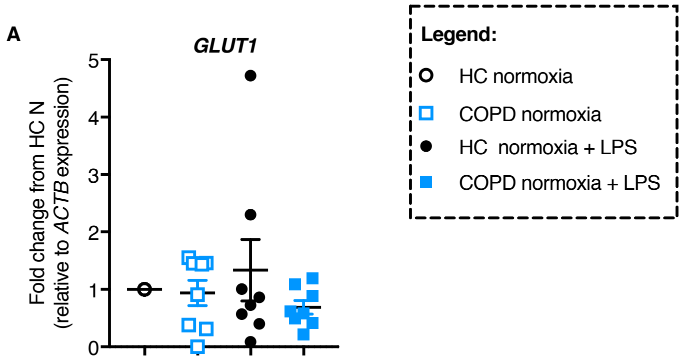

Figure S6. *GLUT1* transcript expression in HC and COPD peripheral blood neutrophils. Related to figure 5

GLUT1 transcript expression in HC and COPD neutrophils cultured for 6 h in the presence and absence of LPS, normalized to β-actin expression. Data are expressed as individual data points with mean +/- SEM, n=8.3

Age (years)	58.7 (40-76)
Gender (M:F)	10:14
FFEV$_1$(L) (forced expiratory volume in 1 second)	1.6 (0.7-2.5)
FEV1% (forced expiratory volume in 1 second %)	60 (33-98)
COPD GOLD stage I:II:III:IV	5:12:4:1
Smoking status (Ex:Current)	11:13
Pack years	40.2 (20-80)

Table S1. COPD patient demographics. Related to figure 5
