# Supplementary material for: Neutrophils Fuel Effective Immune Responses through Gluconeogenesis and Glycogenesis
Source: Cell Metab. 2021 Feb 2;33(2):411–423.e4. doi: 10.1016/j.cmet.2020.11.016 (PMC7863914; doi:10.1016/j.cmet.2020.11.016)
Supplement: Document S2. Article plus Supplemental Information [file mmc2.pdf]

Cell Metabolism

Article

Neutrophils Fuel Effective Immune Responses through Gluconeogenesis and Glycogenesis

Graphical Abstract

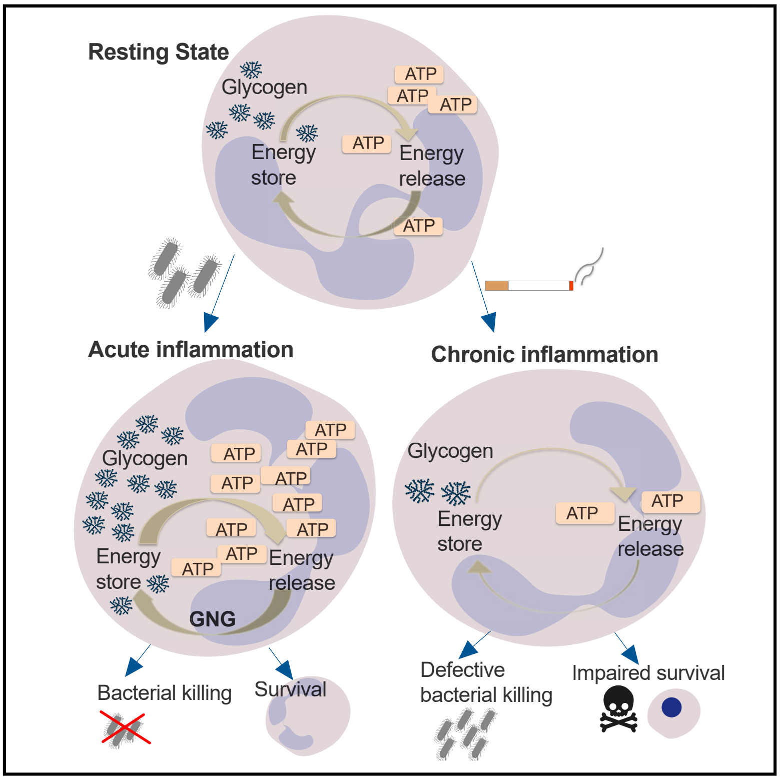

Authors

Pranvera Sadiku, Joseph A. Willson, Eilise M. Ryan, ..., Peter Carmeliet, Moira K.B. Whyte, Sarah R. Walmsley

Correspondence

sarah.walmsley@ed.ac.uk

In Brief

Neutrophils are required to meet their energy demands at inflamed sites where nutrients may be limited. Sadiku et al. provide evidence of a specialized metabolism that enables neutrophils to utilize glycogen cycling for energy production. This is essential for neutrophil function and survival, and dysregulation is associated with chronic disease states.

Highlights

- Neutrophils utilize gluconeogenesis (GNG) to generate glycogen stores
- Glycogenesis is essential for neutrophil survival and function
- Defective glycogen cycling impairs neutrophil function in COPD
- Glycogen cycling underpins the metabolic specialization of neutrophils

Sadiku et al., 2021, Cell Metabolism *33*, 411–423
February 2, 2021 © 2020 The Authors. Published by Elsevier Inc.
https://doi.org/10.1016/j.cmet.2020.11.016

CellPress

Cell Metabolism

CellPress
OPEN ACCESS

Article

Neutrophils Fuel Effective Immune Responses through Gluconeogenesis and Glycogenesis

Pranvera Sadiku,[1,2] Joseph A. Willson,[1] Eilise M. Ryan,[1] David Sammut,[3] Patricia Coelho,[1] Emily R. Watts,[1] Robert Grecian,[1] Jason M. Young,[1] Martin Bewley,[3] Simone Arienti,[1] Ananda S. Mirchandani,[1] Manuel A. Sanchez Garcia,[1] Tyler Morrison,[1] Ailing Zhang,[1] Leila Reyes,[1] Tobias Griessler,[1] Privjyot Jheeta,[1] Gordon G. Paterson,[1] Christopher J. Graham,[1] John P. Thomson,[4] Kenneth Baillie,[5] A.A. Roger Thompson,[3] Jessie-May Morgan,[1] Abel Acosta-Sanchez,[6] Veronica M. Dardé,[6] Jordi Duran,[7,8] Joan J. Guinovart,[7,8,9] Gio Rodriguez-Blanco,[10] Alex Von Kriegsheim,[10] Richard R. Meehan,[4] Massimiliano Mazzone,[11] David H. Dockrell,[1] Bart Ghesquiere,[6] Peter Carmeliet,[2,12,13] Moira K.B. Whyte,[1] and Sarah R. Walmsley[1,14,*]
[1]University of Edinburgh Centre for Inflammation Research, The Queen's Medical Research Institute, University of Edinburgh, Edinburgh EH16 4TJ, UK
[2]Laboratory of Angiogenesis and Vascular Metabolism, Center for Cancer Biology, VIB, Department of Oncology, Leuven Cancer Institute, KU Leuven, Leuven 3000, Belgium
[3]Department of Infection, Immunity and Cardiovascular Disease, University of Sheffield, Sheffield S10 2RX, UK
[4]MRC Human Genetics Unit, Institute of Genetics and Molecular Medicine, University of Edinburgh, Edinburgh EH4 2XU, UK
[5]The Roslin Institute, University of Edinburgh, Easter Bush, Midlothian EH25 9RG, UK
[6]Metabolomics Expertise Centre, VIB-KU Leuven Centre for Cancer Biology, Leuven 3000, Belgium
[7]Institute for Research in Biomedicine (IRB Barcelona), Barcelona Institute of Science and Technology, Barcelona 08028, Spain
[8]Centro de Investigación Biomédica en Red de Diabetes y Enfermedades Metabólicas Asociadas (CIBERDEM), Madrid 28029, Spain
[9]Department of Biochemistry and Molecular Biomedicine, University of Barcelona, Barcelona 08028, Spain
[10]Cancer Research UK Edinburgh Centre, MRC Institute of Genetics and Molecular Medicine, University of Edinburgh, Edinburgh EH4 2XU, UK
[11]Laboratory of Tumor Inflammation and Angiogenesis, VIB-KU Leuven Centre for Cancer Biology, Leuven 3000, Belgium
[12]Laboratory for Translational Breast Cancer Research, Department of Oncology, KU Leuven, Leuven 3000, Belgium
[13]State Key Laboratory of Ophthalmology, Zhongshan Ophthalmic Center, Sun Yat-Sen University, Guangzhou, Guangdong, P.R. China
[14]Lead Contact
*Correspondence: sarah.walmsley@ed.ac.uk
https://doi.org/10.1016/j.cmet.2020.11.016

SUMMARY

Neutrophils can function and survive in injured and infected tissues, where oxygen and metabolic substrates are limited. Using radioactive flux assays and LC-MS tracing with U-^{13}C glucose, glutamine, and pyruvate, we observe that neutrophils require the generation of intracellular glycogen stores by gluconeogenesis and glycogenesis for effective survival and bacterial killing. These metabolic adaptations are dynamic, with net increases in glycogen stores observed following LPS challenge or altitude-induced hypoxia. Neutrophils from patients with chronic obstructive pulmonary disease have reduced glycogen cycling, resulting in impaired function. Metabolic specialization of neutrophils may therefore underpin disease pathology and allow selective therapeutic targeting.

INTRODUCTION

Neutrophils are the most abundant leukocyte, constituting around 60% of the circulating pool. These short-lived cells are the first to accumulate at inflamed tissues where their primary role is to phagocytose and degrade invading pathogens. The requirement to access ATP rapidly to enable an effective host pathogen response places very specific energy requirements upon these cells. In diseases such as chronic obstructive pulmonary disease (COPD), dysfunctional neutrophilic inflammation is associated with impaired pathogen control and prolonged inflammation, which contribute significantly to pathology (Desai et al., 2014; Hogg et al., 2004). Changes in metabolic flux have been associated with murine macrophage polarization and T cell receptor activation, with associated alteration of immune cell function (Mills et al., 2016; Tyrakis et al., 2016). How neutrophils dynamically regulate metabolic pathways to fuel antimicrobial responses in the tissues where nutrient supply may be limited remains an important question in the field.

The existing dogma regarding neutrophil metabolism dates from *in vitro* studies in the 1950s, detailing the importance of glucose-fueled glycolysis for phagocytosis and respiratory burst activity (Borregaard and Herlin, 1982; Sbarra and Karnovsky, 1959). Neutrophils cultured in glucose-enriched media utilized extra-cellular glucose for glycolysis and had very low rates of oxidative phosphorylation, in keeping with observations that

This is an open access article under the CC BY license (http://creativecommons.org/licenses/by/4.0/).

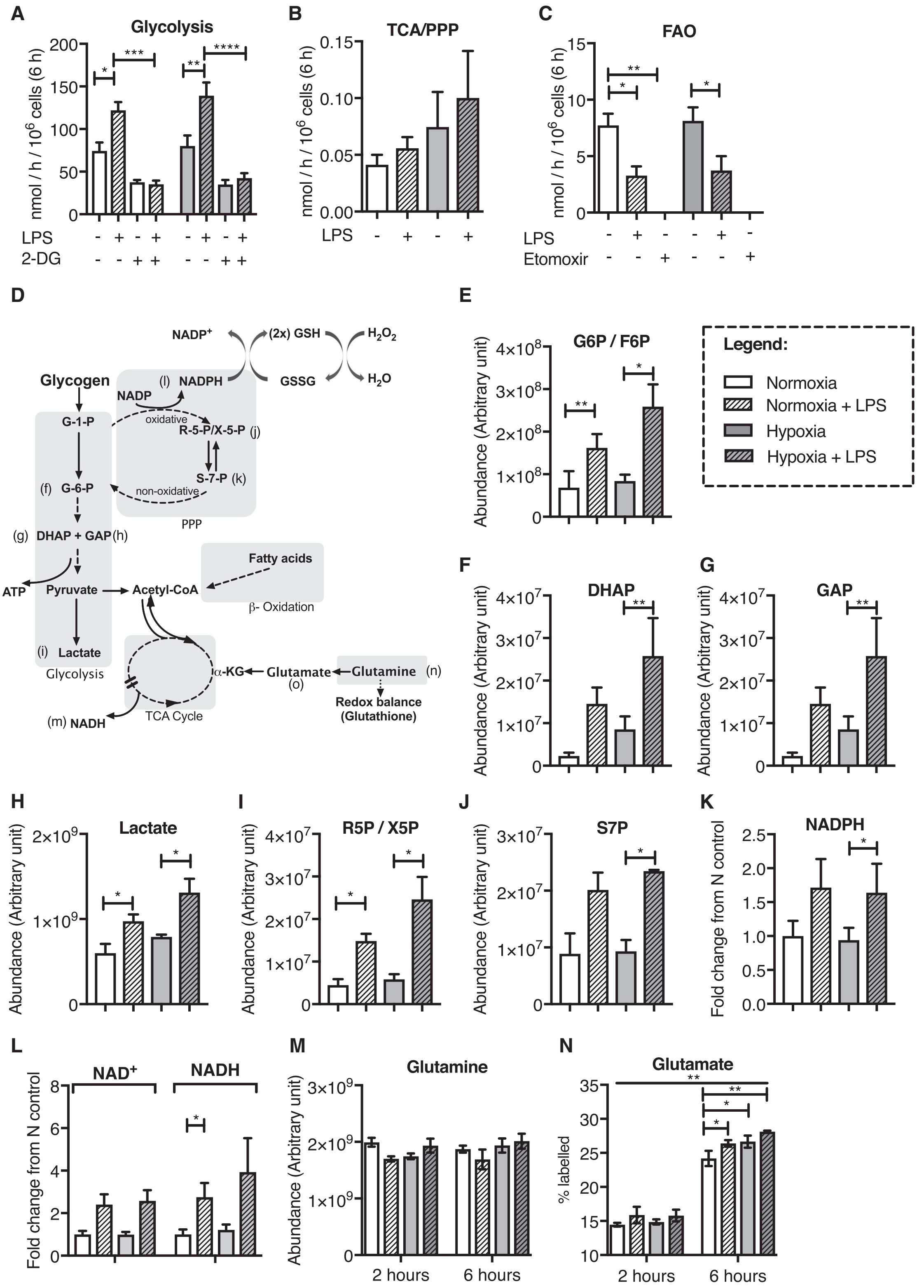

Figure 1. Neutrophil Stimulation Results in Upregulated Glycolytic and PPP Activity, Redox Buffer Capacity, and Glutamine Utilization

(A–C) Radioactive flux assay analysis of human neutrophils following 6 h of culture showing glycolysis (A), TCA/PPP cycle (B), and fatty acid oxidation (FAO) (C) in unstimulated and stimulated neutrophils under normoxic (N, 21% O_2, white bar) and hypoxic (H, 1% O_2, gray bar) culture. (A) N and N + LPS, n = 12; N + 2-DG, n = 6; N + LPS + 2-DG, n = 4; H and H + LPS, n = 11; H + 2-DG, n = 5; H + LPS + 2-DG, n = 3. (B) n = 4 for all conditions. (C) N and N + LPS, n = 6; H and H + LPS, n = 5; N + etomoxir and H + etomoxir, n = 3.

(legend continued on next page)

CellPress
OPEN ACCESS

inhibition of oxidative phosphorylation has little effect on neutrophil oxygen consumption or reactive oxygen species (ROS) production (Fossati et al., 2003), whereas inhibition of glycolysis using 2-deoxyglucose (2-DG) severely impairs their phagocytic and bacterial killing functions (Boxer et al., 1977). More recently, studies of human and murine deficiencies in the G6P transporter and G6Pase-β, respectively, have reported that defective intracellular glucose cycling can also impact key neutrophil functions. In these settings, impaired glucose shuttling between the endoplasmic reticulum and cytoplasm results in neutropenia and defects in respiratory burst activity, ATP production, and bacterial killing (Cheung et al., 2007; Jun et al., 2012; Kim et al., 2008). There is also evidence, however, that a disproportionate increase in glycolysis fuels neutrophil persistence in inflamed airways, resulting in lung injury and death (Sadiku et al., 2017). Neutrophils must therefore tightly regulate glucose utilization and flux through glycolysis to match energy provision to the immediate energy demands of a proportionate but effective host response.

Neutrophil dependence upon glycolysis allows these cells to function and survive at inflamed sites, where limited oxygen availability may render oxidative metabolism ineffective at meeting energy demand. Since extracellular glucose may also be limited at inflamed sites (Garnett et al., 2012; Naughton et al., 1993), we questioned whether neutrophils have a more complex metabolism than previously thought to ensure they can meet energy demand independently of immediate extracellular substrate availability. In this regard, it is interesting to note that neutrophils have been shown to contain glycogen within granules, with early evidence that neutrophil glycogen stores can be modified between circulating and recruited cells, by activation of the oxygen sensing response, and by stimulation with proinflammatory mediators (Robinson et al., 1982; Sadiku et al., 2017; Scott, 1968; Valentine et al., 1953). In this work, we define the metabolic programs that enable neutrophils to generate intra-cellular energy stores and retain function and survival responses under conditions of physiological stress and activation. Further, we demonstrate dysregulation of these metabolic processes in chronic inflammatory disease, with consequences for neutrophil function and survival.

RESULTS

Pro-inflammatory Mediators Increase Neutrophil Glycolytic Flux, Redox Buffering Capacity, and Glutamine Utilization

We used radioactively labeled substrates to characterize which metabolic pathways neutrophils utilize in basal and LPS-stimulated states under conditions of both normoxia and hypoxia (Figures 1A–1C). In keeping with the literature, we observed unstimulated neutrophils to be highly glycolytic, with equivalent glycolytic capacity in normoxia and hypoxia (Figure 1A). LPS stimulation significantly increased glycolytic flux, and this could be reversed by inhibition of glycolysis with 2-DG (Figure 1A). Although detectable levels of glucose oxidation and pentose phosphate pathway (PPP) activity were seen, these were 100-fold less than flux observed through glycolysis (Figure 1B). While at baseline neutrophils had a relatively high level of fatty acid oxidation (FAO), this was suppressed by LPS stimulation (Figure 1C). Metabolic pathways required for ATP and redox are summarized in Figure 1D. To more specifically delineate the effects of physiological stressors on these pathways, we undertook liquid chromatography-mass spectrometry (LC-MS) quantification of individual metabolic intermediaries. We observed that LPS increased relative abundance of key glycolytic metabolites, glucose-6-phosphate/fructose-6-phosphate (Figure 1E), dihydroxyacetone phosphate (DHAP) (Figure 1F), glyceraldehyde-3-phosphate (Figure 1G), and lactate (Figure 1H). In addition, levels of the PPP substrates ribose-5-phosphate (Figure 1I) and sedoheptulose-7-phosphate (Figure 1J), an important branch pathway of glycolysis that enables neutrophil respiratory burst activity (Bender and Van Epps, 1985), were also induced in response to LPS. In keeping with the increased flux through the PPP and therefore redox buffering capacity, LPS stimulation also significantly increased levels of NADPH and NADH under hypoxic culture (Figures 1K and 1L). Given that neutrophil supplementation with glutamine has previously been described to promote neutrophil phagocytosis, we also analyzed glutamine availability in un-stimulated and stimulated neutrophils under hypoxic and normoxic culture. Although we were unable to detect a significant increase in the total abundance of glutamine in response to LPS (Figure 1M), we found that LPS upregulated glutamine conversion into glutamate (Figure 1N), indicative of increased glutamine utilization in LPS-stimulated neutrophils.

Favorable Neutrophil Energetics Are Maintained by Glycolysis under Conditions of Physiological Stress

Radioactive flux assay data were used to predict the relative contribution of FAO, oxidative phosphorylation via the tricarboxylic acid (TCA) cycle, and glycolysis to neutrophil production of ATP (De Bock et al., 2013). These calculations supported glycolysis contributing a significantly higher proportion of ATP production to neutrophils than FAO under both normoxic and hypoxic culture and following LPS stimulation (Figures 2A and 2B). To validate these calculations, we measured the energy status of neutrophils (ATP/ADP ratio) in the presence of chemical inhibitors of glycolysis (2-DG) (Figure 2C), FAO (etomoxir) (Figure 2D), and oxidative phosphorylation (oligomycin A) (Figure 2E)

(D) A pathway diagram showing the metabolites measured in human neutrophils following 6 h of culture in normoxia and hypoxia in the presence or absence of LPS stimulation.

(E–L) LC-MS analyses of the neutrophil intracellular abundance of glycolytic metabolites (glucose-6-phosphate/fructose-6-phosphate, G6P/F6P; dihydroxyacetone phosphate, DHAP; glyceradehyde-3-phosphate, GAP; lactate), pentose phosphate pathway metabolites (PPP; ribose-5-phosphate/xylulose-5-phosphate, R5P/X5P; sedoheptulose-7-phosphate, S7P), and redox buffers (NADPH and NADH). n = 3.

(M and N) LC-MS time course analyses of the neutrophil intracellular levels of amino acid glutamine (M; n = 3) and percentage heavy labeled glutamate (N; n = 3) following culture with U-^{13}C glutamine.

Data represent mean ± SEM. p values obtained via unpaired t tests (A–C), paired t tests (D–L), or two-way ANOVA with Tukey's multiple comparisons test; overall significance shown for increase in labeled glutamate from 2 to 6 h (N). *p < 0.05, **p < 0.01, ***p < 0.005, ****p < 0.001.

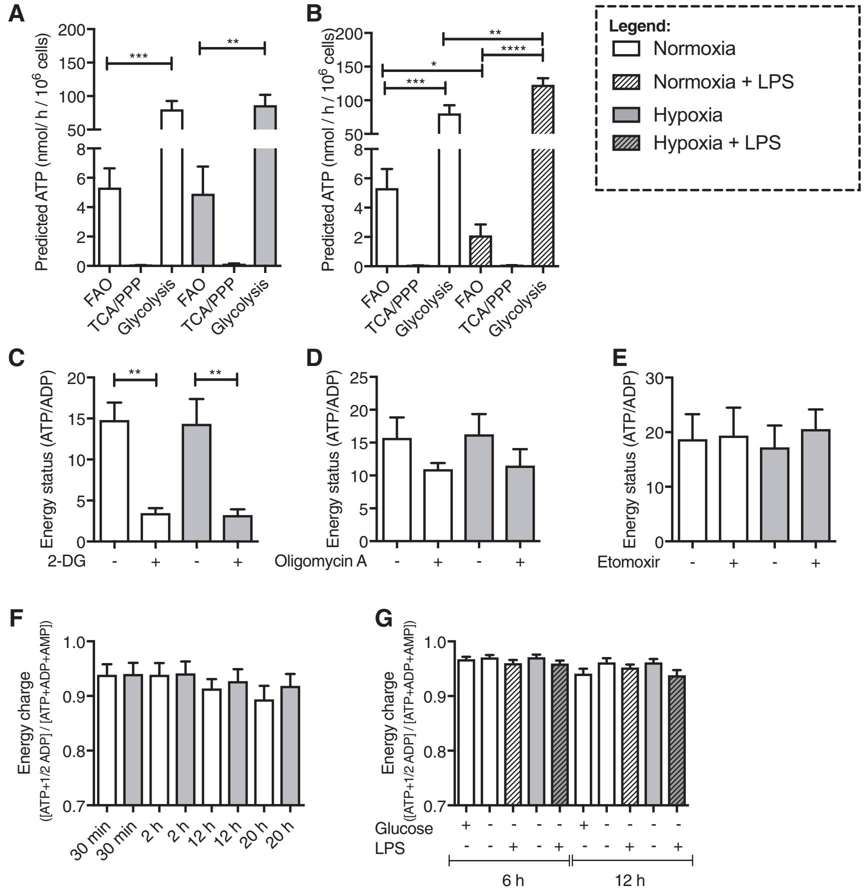

Figure 2. Neutrophil ATP Is Predominantly Generated by Glycolysis with Maintenance of Energy Charge during Neutrophil Lifespan and in a Glucose-Deplete Setting

(A and B) ATP contribution of fatty acid oxidation (FAO), tricarboxylic acid cycle (TCA), and glycolysis were derived from calculation from the radioactive flux assays comparing normoxia to hypoxia (A) and unstimulated to LPS-stimulated neutrophils following 6 h of culture (B). Normoxia, FAO, n = 6; TCA, n = 3; glycolysis, n = 9; hypoxia, FAO, n = 4; TCA, n = 3; glycolysis, n = 8: normoxia + LPS, FAO, n = 6; TCA, n = 3; glycolysis, n = 9.

(C–E) Energy status measurement (ATP/ADP) of neutrophils cultured for 2 h in the presence and absence of pathway inhibitors 10 mM 2-DG (C; n = 4), 1.2 μM oligomycin A (D; n = 3), and 10 μM etomoxir (E; n = 3).

(F and G) Energy charge measurement ([ATP + 1/2ADP]/[ATP + ADP + AMP]) in neutrophils cultured for 2, 6, and 20 h in normoxia and hypoxia (F) and for 12 h in glucose-replete and -deplete medium in unstimulated and LPS-stimulated cells (G). (F) normoxia + LPS-glucose, n = 4; hypoxia + glucose, n = 3. (G) n = 3.

Data represent mean ± SEM. p values obtained via unpaired t test (A and B) or paired t test (C). *p < 0.05, **p < 0.01, ***p < 0.005, ****p < 0.001.

(Neubert and Lehninger, 1962; Pike et al., 2011; Wick et al., 1957). Only treatment of neutrophils with the hexokinase inhibitor 2-DG resulted in a failure of neutrophils to maintain their high energy status, independent of oxygen availability (Figures 2C–2E). It is important to note that 2-DG may be exerting off-target effects through inhibition of N-glycosylation and induction of the unfolded protein response (Yu and Kim, 2010). To assess whether neutrophils have the capacity to actively maintain favorable energetics, we measured relative levels of ATP, ADP, and AMP over time, and determined the energy charge ([ATP + 1/2ADP]/[ATP + ADP + AMP]) of neutrophils under conditions of hypoxia, glucose deprivation, and LPS stimulation. Neutrophils were able to maintain their energetic balance across multiple time points (Figure 2F) even when oxygen and glucose were limited (Figures 2G and S1A–S1C). Neutrophils are therefore adept at maintaining intracellular energy homeostasis both in quiescent and activated states, throughout their lifespan, and despite the presence of physiological stresses including glucose and oxygen deprivation, provided they can maintain an active glycolytic pathway.

Intracellular Glycogen Stores Are Essential for Neutrophil Survival

Having demonstrated neutrophil reliance on glycolysis, even in the absence of extra-cellular glucose, we next questioned whether neutrophils can meet their energy demands by generating and utilizing intracellular glucose stores in the form of glycogen. Glycogen levels in unstimulated and LPS-stimulated cells were measured under conditions of normoxia and hypoxia. LPS-stimulated neutrophils had significantly higher levels of glycogen in both normoxia and hypoxia (Figure 3A). Inhibiting glycogen degradation using the glycogen phosphorylase inhibitor CP-91149 (Martin et al., 1998) increased intracellular glycogen content (Figure 3B), and the resulting inability of neutrophils to access glycogen stores led to impaired neutrophil survival in glucose-deprived cultures of both non-LPS- and LPS-treated cells (Figure 3C).

Neutrophils Use Gluconeogenesis to Generate Glycolytic Intermediaries from Non-glucose Substrates to Increase Their Glycolytic Capacity

Combining the ability of neutrophils to fuel glycolysis and maintain energy states in glucose-deplete conditions with their ability to regulate intracellular glycogen led us to question whether neutrophils can utilize glucose and non-glucose substrates to generate glycogen stores. We first verified that neutrophils regulate glycogenesis by demonstrating increased uptake of U-^{14}C glucose and higher levels of radioactively labeled glycogen following LPS challenge (Figure 3D). Importantly, when neutrophils were cultured with U-^{14}C lactate, we were again able to detect significant levels of radioactivity in the purified glycogen (Figure 3E), indicating that neutrophils can use non-glucose substrates to generate glycogen stores. In keeping with the ability of neutrophils to undergo glycogenesis and gluconeogenesis, we observed that neutrophils express key enzymes required for active glycogenesis (GYS1, GBE1, and UGP2) and gluconeogenesis (FBP1 and PEPCK2) at both mRNA and protein levels (Figures 3F and 3G).

CellPress
OPEN ACCESS

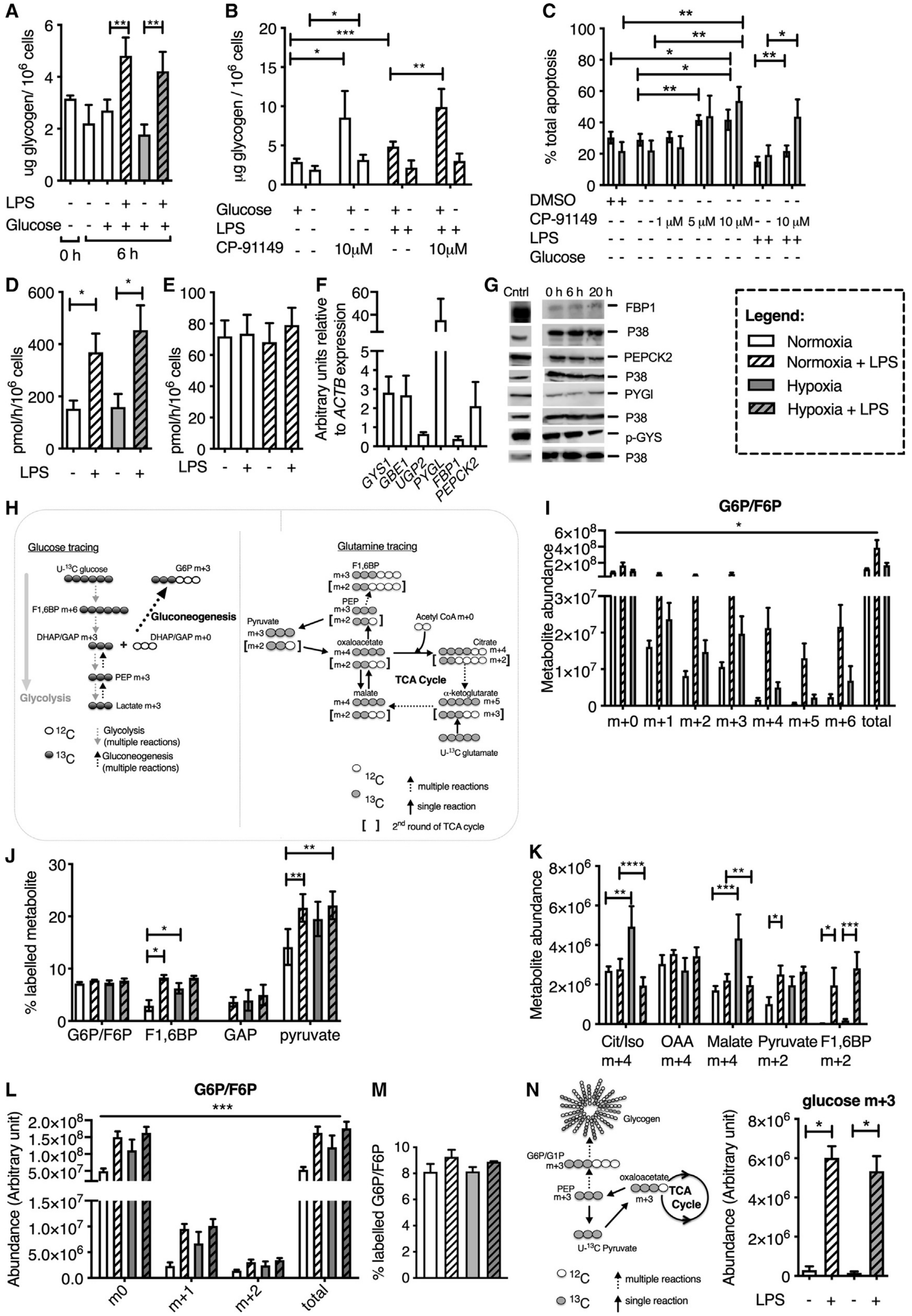

(legend on next page)

To confirm that neutrophils have the capacity for gluconeogenesis, cells were incubated with heavy U-^{13}C glucose, U-^{13}C glutamine, and U-^{13}C palmitate tracers, and analysis of ^{13}C labeling was quantified by LC-MS. A schematic detailing the putative entry and cycling of carbon molecules from U-^{13}C glucose (black circles) and U-^{13}C glutamine (gray circles) into TCA and glycolytic isotopologues is provided in Figure 3H. Detection of multiple isotopologues of glucose-6-phosphate/fructose-6-phosphate following neutrophil culture with U-^{13}C glucose (Figure 3I) and the presence of heavy carbons within glycolytic and TCA intermediaries following neutrophil culture with U-^{13}C glutamine (Figures 3J–3L) and U-^{13}C palmitate (Figure 3M) together provide definitive evidence for active neutrophil gluconeogenesis. The ability of neutrophils to induce gluconeogenic activity was observed with both LPS treatment (Figures 3I–3L) and in hypoxia (Figures 3J–3L), with higher levels of labeled carbons from glutamine observed in glycolytic intermediaries (Figure 3J). Thus, glutamine contributes to the increase in gluconeogenesis observed in response to hypoxia and LPS. The full isotopologue profiles for citrate/isocitrate, oxaloacetate, malate, pyruvate, and F1,6BP following neutrophil culture for 4 h with U-^{13}C glutamine are shown in Figures S2A–S2F, with significant differences in substrate utilization observed within 2 h of U-^{13}C glutamine supplementation (Figures S3A–S3F). Increased tracing of U-^{13}C glutamine carbons into PPP metabolites was also observed with LPS stimulation (Figures S4A and S4B). In order to directly assess whether gluconeogenesis contributes to the synthesis of glycogen, we undertook the tracing of heavy carbons from pyruvate into glycogen. ^{13}C-labeled glucose generated from hydrolyzed glycogen was observed in LPS-stimulated neutrophils (Figure 3N), providing direct evidence for a role of gluconeogenesis in fueling glycogen synthesis.

Glycogen Storage Capacity Dictates Neutrophil Function and Survival

With evidence for dynamic regulation of both gluconeogenesis and glycogenesis, we questioned the interplay between these pathways and their importance for neutrophil function and survival. Culture of LPS-stimulated neutrophils with the glutaminase inhibitor BPTES significantly reduced intracellular glycogen levels, supporting a role for glutaminolysis in the synthesis of glycogen (Figure 4A). Furthermore, neutrophil intracellular glycogen levels were also significantly reduced following inhibition of gluconeogenesis (FBP1) with the inhibitor MB05032 (Figure 4B). The functional consequence of limiting glutamine access and blocking gluconeogenesis was next explored. Glutamine deprivation and inhibition of either PEPCK (3-mercaptopicolinic acid, 3-MPA) or FBP1 (FBPi or MB05032) resulted in reduced neutrophil survival (Figures 4C–4E). Neutrophils also displayed defective bacterial killing when gluconeogenesis (Figure 4F) and glutaminolysis (Figure 4G) were disrupted. To directly test whether gluconeogenesis and glycogen shuttling regulate neutrophil function *in vivo*, we generated a neutrophil-specific glycogen synthase (*Gys1*) knockout mouse (Figures S5A and S5B). Targeting GYS1, the dominant isoform in neutrophils, enabled us to prevent the generation of glycogen in neutrophils, therefore abrogating the glycogen shuttle. Gys1^{loxlox}MRP8-cre$^{+/-}$ mice demonstrated no baseline defect in blood neutrophil numbers (Figure S5C) or monocyte proportion (Figure S5D). Airspace neutrophils were isolated from mice 24 h post-LPS challenge, and the consequences of GYS1 loss for apoptosis and bacterial killing were assessed *ex vivo*. In order to identify the neutrophil niche in which glycogen is most important, glycogen was first measured in bone marrow (BM), blood, and bronchoalveolar lavage (BAL) neutrophils. We found that glycogen levels in the BM and circulating neutrophils of naive and LPS-challenged wild-type (WT) mice are significantly lower than those of BAL neutrophils recruited following LPS challenge (Figure 4H). Subsequent assessment of glycogen content in blood and airspace neutrophils from Gys1^{loxlox}MRP8-cre$^{+/-}$ and Gys1^{loxlox}MRP8-cre$^{-/-}$ mice 24 h post-LPS challenge confirmed that *Gys1* loss results in a significant reduction in levels of glycogen in these cells (Figure 4I). *In vitro*, this translated into functional consequences shown by an increase in apoptosis

Figure 3. Regulation of Glycogen Stores and Gluconeogenesis Pathway Activity during Human Neutrophil Activation

(A) Glycogen level quantification in freshly isolated neutrophils (0 h) and following 6 h of culture in glucose-deplete and -replete media under normoxia and hypoxia. n = 4.

(B) Glycogen content of neutrophils following 6 h of normoxic culture with the glycogen phosphorylase inhibitor CP-91149 preventing glycogen breakdown. n = 4.

(C) Assessment of apoptosis rates using flow cytometry following culture in glucose-deprived media under normoxia and hypoxia with CP-91149 and LPS for 12 h. n = 5.

(D and E) Liquid scintillation count measurement of radioactive U-^{14}C glucose (D; n = 4) and U-^{14}C lactate (E; n = 5) incorporation into neutrophil glycogen stores following 6 h of culture.

(F) Transcript expression of glycogen metabolism and gluconeogenesis machinery: muscle glycogen synthase (*GYS1*, n = 4), glycogen branching enzyme (*GBE1*, n = 4), UDP-glucose pyrophosphorylase 2 (*UGP2*, n = 3), liver glycogen phosphorylase (*PYGL*, n = 4), fructose-1,6-bisphosphatase 1 (*FBP1*, n = 4), and phosphoenolpyruvate carboxykinase 2 (*PEPCK2*, n = 4).

(G) Protein expression of glycogen metabolism and gluconeogenesis machinery in freshly isolated neutrophils (0 h) and neutrophils cultured for 6 or 20 h. Positive controls for FBP1 and PEPCK2− MCF7 lysate, PYGL− mouse liver lysate, phospho-GYS (p-GYS), and GYS− NIH/3T3 cell lysate. Representative western blots are shown. n = 3.

(H) Diagrammatic representation of U-^{13}C glucose (black circles) and U-^{13}C glutamine (gray circles) labeling in human neutrophils. GNG, gluconeogenesis.

(I) G6P/F6P isotopologue abundance following culture in U-^{13}C glucose media for 4 h under conditions of normoxia, normoxia with LPS, and hypoxia. n = 4.

(J–L) ^{13}C percentage labeling of glycolytic intermediaries (J; n = 4) and isotopologue labeling of TCA cycle and glycolytic intermediaries (K and L; n = 4) following 4 h of culture in U-^{13}C glutamine containing media.

(M) Percentage heavy labeling of G6P/F6P following 4 h of culture in the presence of U-^{13}C palmitic acid. n = 4.

(N) Schematic diagram and relative abundance of glucose m+3 isotopologue following U-^{13}C pyruvate tracing in neutrophils derived by LC-MS analysis of hydrolyzed glycogen. n = 4.

Data represent mean ± SEM. Statistical significance was determined by paired t tests (A, B, and D) or two-way ANOVA with Tukey's multiple comparisons test (C and J–L) and a one-way ANOVA with Tukey's multiple comparisons test (N). *p < 0.05, **p < 0.01, ***p < 0.005. (J) Significance shown for unstimulated versus LPS stimulated cells across all isotopologues.

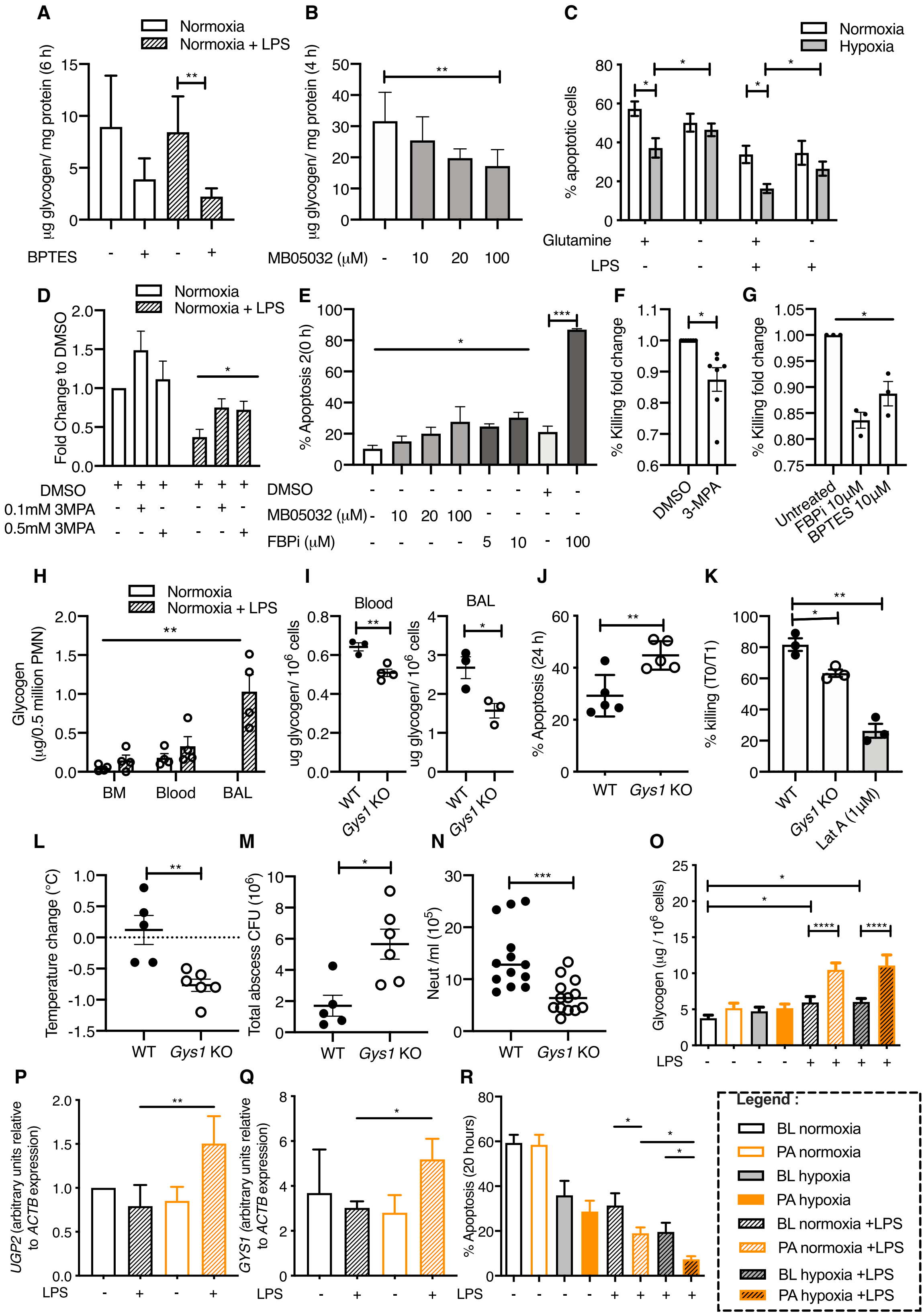

(legend on next page)

(Figure 4J) and reduced bacterial killing (Figure 4K). Importantly, Gys1^{loxlox}MRP8-cre$^{+/-}$ mice revealed an impaired host response following *in vivo* challenge with subcutaneous administration of *Staphylococcus aureus* (Figures 4L–4N). Systemically, these mice developed a significant degree of hypothermia (Figure 4L), with increased bacterial counts within the skin abscess 24 h following bacterial challenge (Figure 4M), and reduced circulating neutrophil and monocyte numbers (Figures 4N and S5E).

With evidence that glycogen shuttling is important for neutrophil-mediated host responses, we questioned whether changes in glycogen stores had long-term consequences for neutrophil survival. Recent work from our group has shown that sustained exposure of mice to systemic hypoxia reprograms neutrophil glucose utilization (Thompson et al., 2017), and that activation of oxygen sensing responses can alter neutrophil glycogen stores (Sadiku et al., 2017). We therefore hypothesized that exposure of healthy human volunteers to altitude-induced hypoxia would enhance the capacity for activated neutrophils to generate glycogen stores with long-term consequences for neutrophil survival. Twelve healthy human volunteers were exposed to 7 days of altitude-induced hypoxia (Figures S5F and S5G) and their peripheral blood neutrophils were isolated at baseline (BL), at altitude, and 3 months post altitude (PA), then cultured in the presence or absence of LPS. Altitude exposure resulted in significantly increased neutrophil glycogen stores following LPS stimulation (Figure 4O), driven by induction of UGP2 and glycogen synthase GYS1 (Figures 4P, 4Q, S5H, and S5I). This inflammatory uplift in glycogen stores following altitude exposure was paralleled by enhanced neutrophil survival responses when compared to baseline (Figures 4R, S5K, and S5L).

Defective Induction of Gluconeogenesis and Reduced Glycogen Cycling Impairs Neutrophil Function in COPD

Disordered neutrophilic inflammation is a characteristic feature of COPD, with bacterial persistence and ongoing neutrophil recruitment associated with more rapid disease progression. A disconnect between high neutrophil numbers and failed pathogen clearance in this setting is indicative of aberrant neutrophil function, a phenotype of considerable therapeutic interest. Peripheral blood neutrophils from patients with GOLD stage 1–4 COPD (Table S1) and no evidence of systemic hypoxia and respiratory failure were studied. Patient neutrophils had impaired bacterial killing of *Streptococcus pneumoniae* S14 (Figure 5A) and *S. aureus* SH1000 (Figure 5B), with defective LPS survival responses (Figure 5C). Importantly, impaired survival responses could not be rescued by glucose supplementation, indicating an intrinsic neutrophil defect independent of glucose availability. In keeping with a dysregulated metabolic status, COPD neutrophils had significantly lower ATP levels at baseline when compared to healthy controls (Figure 5D) and a reduced glycolytic response to SH1000 (Figures 5E and 5F). Diminished glycolytic capacity occurred despite preservation of glucose uptake with equivalent tracing of U-^{13}C glucose into glucose-6-phosphate (Figure 5G) and retained expression of the dominant neutrophil glucose transporter GLUT1 (Figure S6A) in unstimulated and LPS-stimulated states. Extending our observations that neutrophils have an active gluconeogenesis pathway that uses non-glucose substrates to generate glycogen, and the importance of glycogen stores for neutrophil survival, we therefore questioned whether defective gluconeogenesis and glycogen cycling could result in these dysfunctional neutrophil responses. Initial baseline phenotyping of peripheral blood neutrophils revealed an impaired ability of COPD neutrophils to increase glycogen levels in response to LPS (Figure 5H) and to increase transcription of the key gluconeogenic enzyme PEPCK2 (Figure 5I) and the glycogenic enzyme GBE1 (Figure 5J). LC-MS analysis of healthy control and COPD blood neutrophils was then undertaken to delineate the metabolic consequences of impaired glycogen cycling in these cells. COPD neutrophils displayed significantly lower lactate levels with normoxic culture alone and following LPS stimulation (Figure 5K). This was associated with a decreased level of lactate in the medium of COPD neutrophils cultured with LPS (Figure 5L) and reduced levels of the PPP substrates ribose-5-phosphate (Figure 5M) and sedoheptulose-7-phosphate (Figure 5N). COPD neutrophils also demonstrated a failure to generate the metabolites required for effective redox buffering capacity (NADH) (Figure 5O)

Figure 4. Glycogen Storage Capacity Dictates Neutrophil Function and Survival in Gys1^{loxlox}MRP8-cre$^{+/-}$ Mice and Healthy Human Volunteers Exposed to Altitude-Induced Hypoxia

(A and B) Neutrophil glycogen content following 6 (A) or 4 h (B) normoxic culture in glucose-deplete media with the glutaminase inhibitor BPTES preventing glutamine breakdown (A; n = 4) and the gluconeogenesis inhibitor MB05032 (B; n = 3).

(C–E) Assessment of apoptosis rates using flow cytometry (C) and cellular morphology (D and E) following culture in glutamine-deprived (C) or glucose-deplete media (D and E) under conditions of normoxia and hypoxia ± LPS for 6 (D) and 20 h (C). Data shown as mean ± SEM (C and D) and fold change from DMSO vehicle control. n = 4.

(F and G) Neutrophils were challenged with *S. aureus* (SH1000) at a multiplicity of infection (MOI) of 10 and bacterial killing assessed by flow cytometry. Data shown as fold change from DMSO (F; n = 7) and untreated (G; n = 3) controls.

(H) Glycogen content of neutrophils in the bone marrow, blood, and bronchoalveolar lavage (BAL) of untreated and 24 h post-LPS challenge of wild-type mice (WT). n = 3.

(I) Glycogen content of circulating and BAL neutrophils of WT and *Gys1* knockout mice. n = 3.

(J) Assessment of apoptosis rates using flow cytometry following 24 h of culture under standard media conditions. n = 5.

(K) *In vitro* challenge of BAL neutrophils with *S. aureus* (SH1000) (MOI:10) and bacterial killing assessed by flow cytometry. n = 3.

(L–N) *Gys1*$^{lox/lox}$ MRP8-Cre$^{+/-}$ knockout (*GYS1* KO) and *Gys1*$^{lox/lox}$ MRP8-Cre$^{-/-}$ WT mice were inoculated with 5 × 10^7 CFU of *S. aureus* (SH1000) and rectal temperatures (L), total abscess CFU counts (M), and blood neutrophil counts (N) obtained 24 h post-subcutaneous infection.

(O–R) Intracellular glycogen levels (O; n = 6), UGP2 (P; n = 8) and GYS1 (Q; n = 7) relative transcript abundance, and apoptosis rates (R; n = 8) were measured in blood neutrophils isolated from healthy human volunteers at baseline (BL) and 3 months post-altitude-induced hypoxia (PA), following culture *ex vivo* with LPS and hypoxia.

Data represent mean ± SEM. Statistical significance was determined by paired t tests (A, B, D–F, K, P, and Q), two-way ANOVA with Sidak's multiple comparisons test (C, O, and R), one-way ANOVA with Tukey's multiple comparisons test (C, G, and H), and unpaired t tests (I, J, and L–N). *p < 0.05, **p < 0.01, ***p < 0.005, ****p < 0.001.

CellPress
OPEN ACCESS

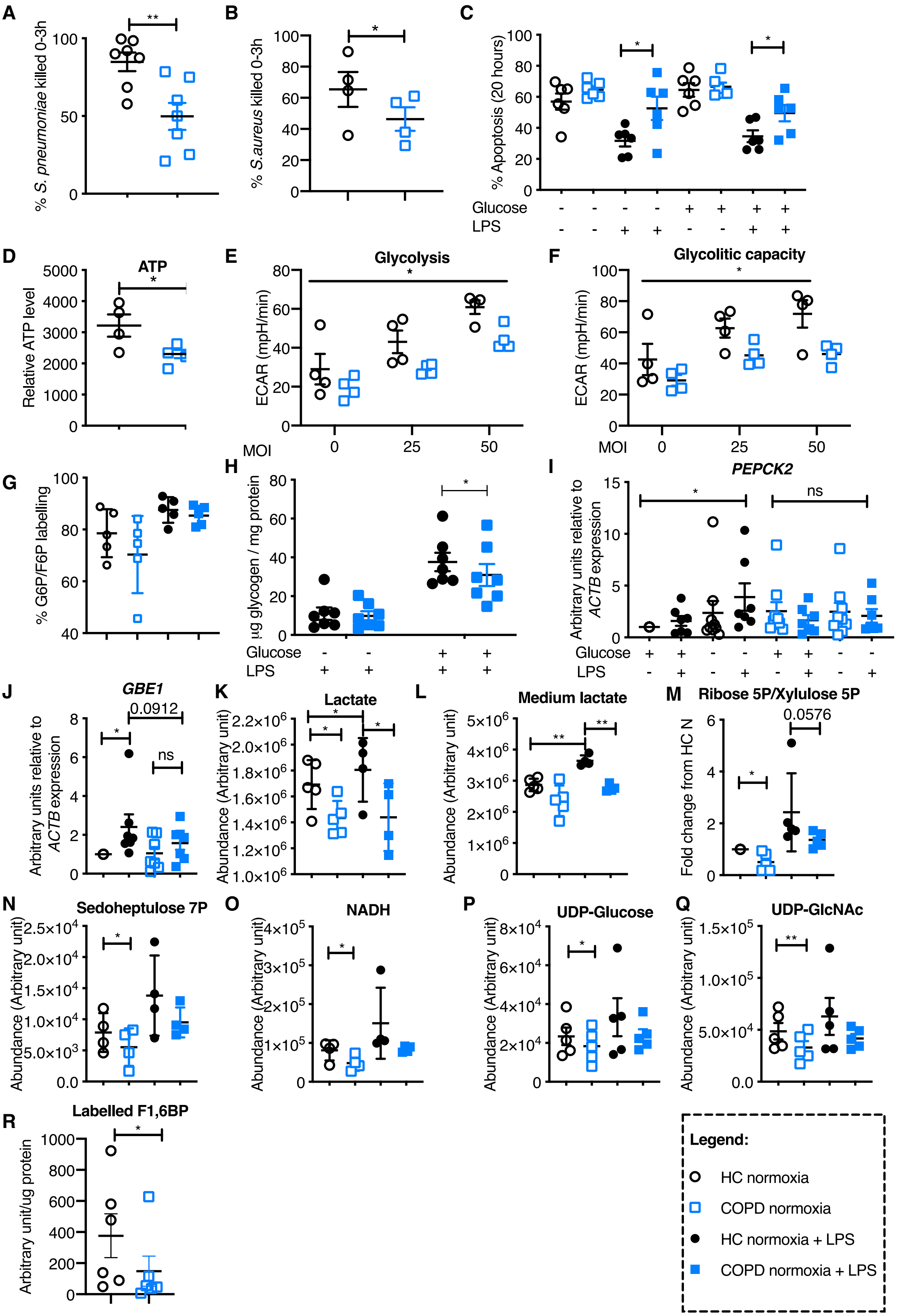

(legend on next page)

and pathogen sensing responses (UDP-glucose and UDP-GlcNAc) (Figures 5P and 5Q) (Jha et al., 2015). Finally, tracing of U-^{13}C glutamine into F1,6BP (Figure 5R) demonstrated that COPD neutrophils have a diminished capacity to utilize glutamine for gluconeogenesis.

Figure 6 summarizes the dynamic balance between neutrophil energy utilization and storage driven by intracellular glycogen stores in quiescent and activated blood neutrophils, and how this becomes uncoupled in chronic inflammatory states.

DISCUSSION

Cellular adaptation to physiological stresses encountered within inflamed tissues is critically dependent upon metabolic responses that maintain ATP and modify energy requirements. Neutrophils are unusual in that they must rapidly access ATP to enable responses, including migration, pathogen control, and apoptosis, that have significant energy requirements (Borregaard and Herlin, 1982). As such, they have evolved a dependence upon glycolysis for ATP production. We have delineated the metabolic processes that enable this metabolic specialization, allowing successful matching of energy supply and demand through the ability of neutrophils to regulate autocrine energy stores in the form of glycogen. Through cycling between gluconeogenesis and glycogenesis, we propose that neutrophils are able to maintain their energy charge throughout their lifespan, even when glucose is limited.

Initial experiments detailing the existence of multiple glucose isotopologues with U-^{13}C glucose tracing, and demonstrating neutrophil expression of unidirectional enzymes required for gluconeogenesis (PCK2 and FBP1), led us to propose that neutrophils can undergo gluconeogenesis. This was verified using LC-MS tracing of heavy carbons from U-^{13}C glutamine and palmitic acid into glycolytic intermediaries and finally U-^{13}C pyruvate into glycogen. The ability of neutrophils to scavenge glutamine for generation of glycolytic intermediaries is particularly important, given the relative availability of these metabolic substrates at the inflamed site, as this provides a mechanism whereby neutrophils can continue to meet energetic demands in the absence of extracellular glucose. The capacity for limited non-hepatic gluconeogenesis is not entirely without precedent. In a glucose-deprived setting, cancer cells have the ability to switch to using glutamine as a substrate and engage the early steps of gluconeogenesis through regulation of mitochondrial PCK2 (Vincent et al., 2015). More recently, maintenance of CD8 T cell memory has been linked to activation of the cytosolic form of this enzyme PCK1 (Ma et al., 2018; Vincent et al., 2015), while loss of PCK1 has also been associated with a reduction in TCA cycle intermediaries and pro-inflammatory macrophage polarization. There is, however, no evidence to date suggesting that myeloid populations other than neutrophils have the capacity to undergo gluconeogenesis (Ko et al., 2018) and only a single report demonstrating the differential regulation of glycogen and glucose metabolism in dendritic cells (Thwe et al., 2017), indicating cell-type-specific metabolic adaptations within the innate immune response and thus the opportunity for neutrophil-specific therapeutic intervention.

We subsequently questioned how neutrophils balance energy utilization with energy storage and the consequences for immune cell function. This was explored by exposing peripheral blood neutrophils from healthy volunteers to differing physiological stresses (glucose depletion and hypoxia), pro-inflammatory activation with LPS, and inhibitors of glycogen degradation. Increased generation of glutamine-derived glycolytic intermediaries together with increased gluconeogenesis fueled glycogen deposition following LPS stimulation of neutrophils would suggest that in response to a proinflammatory stimulus neutrophils increase cycling through gluconeogenesis and glycogenesis to maintain glycogen storage, which in turn fuels the higher energy demands of a proinflammatory response.

Importantly, this glycogen/glucose axis is both amenable to modulation and regulates neutrophil function. Exposing healthy volunteers to altitude-induced hypoxia resulted in augmented intracellular glycogen reserves in response to LPS and enhanced neutrophil survival. The ability of cells to shunt glucose into glycogen has previously been observed in brain astrocytes and cancer cells (Favaro et al., 2012; Obel et al.,

Figure 5. COPD Peripheral Blood Neutrophils Are Unable to Regulate Their Glycogen Synthesis, Resulting in Diminished Intracellular Glycogen Stores, Defective Bacterial Killing, and Survival

(A–C) Neutrophils from healthy control subjects (HC; open black circle) or patients with COPD (COPD; open blue square) were challenged with either opsonized serotype 14 *Streptococcus pneumoniae* (S14) (A; n = 7) or *Staphylococcus aureus* (SH1000) (B; n = 4), at a multiplicity of infection (MOI) of 10 and bacterial killing assessed. Assessment of apoptosis by annexin V/TO-PRO-3 staining of HC and COPD neutrophils following 20 h culture (C; n = 6).

(D) Relative of ATP abundance of freshly isolated neutrophils from HC and COPD using RP-HPLC (reversed-phase high-performance liquid chromatography). HC, n = 4; COPD, n = 6.

(E and F) Seahorse quantification of ECAR of healthy and COPD peripheral blood neutrophils exposed to SH1000 at an MOI of 10, 25, and 50. n = 4.

(G) U-^{13}C glucose incorporation into glucose-6-phosphate/fructose-6-phosphate (G6P/F6P) following 4 h of culture in U-^{13}C glucose containing media under normoxia in the presence (filled symbol) and absence (open symbol) of LPS. n = 5.

(H) Glycogen content of HC and COPD neutrophils cultured in glucose-replete and glucose-deplete media for 6 h in the presence and absence of LPS. n = 7.

(I and J) Relative transcript abundance of the gluconeogenic gene *PEPCK2* (I; n = 7, unstimulated; n = 9, LPS) and glycogen synthesis pathway gene *GBE1* (J; n = 7) in HC and COPD neutrophils cultured for 6 h in the presence and absence of LPS normalized to β-actin expression.

(K–Q) Healthy control and COPD peripheral blood neutrophils were cultured in U-^{13}C glucose containing media for 4 h under normoxia in the presence and absence of LPS. Total intracellular lactate (K; n = 5), medium lactate (L; n = 5), ribose5P/xylulose5P (M; n = 5), sedoheptulose7P (N; n = 5), NADH (O; n = 4), UDP-glucose (P; n = 5), and UDP-GlcNAc (Q; n = 5) were measured using LC-MS and normalized to protein content. Fold change was determined relative to the paired unstimulated HC control (M).

(R) Healthy control and COPD peripheral blood neutrophils were cultured in U-^{13}C glutamine containing media for 4 h under normoxia and total labeled F1,6BP measured using LC-MS. n = 6.

Data are expressed as individual data points with mean ± SEM. Statistical significance was determined by paired t tests (A–D and H–R) or two-way ANOVA with Sidak's multiple comparisons test (E and F). *$p < 0.05$, **$p < 0.01$.

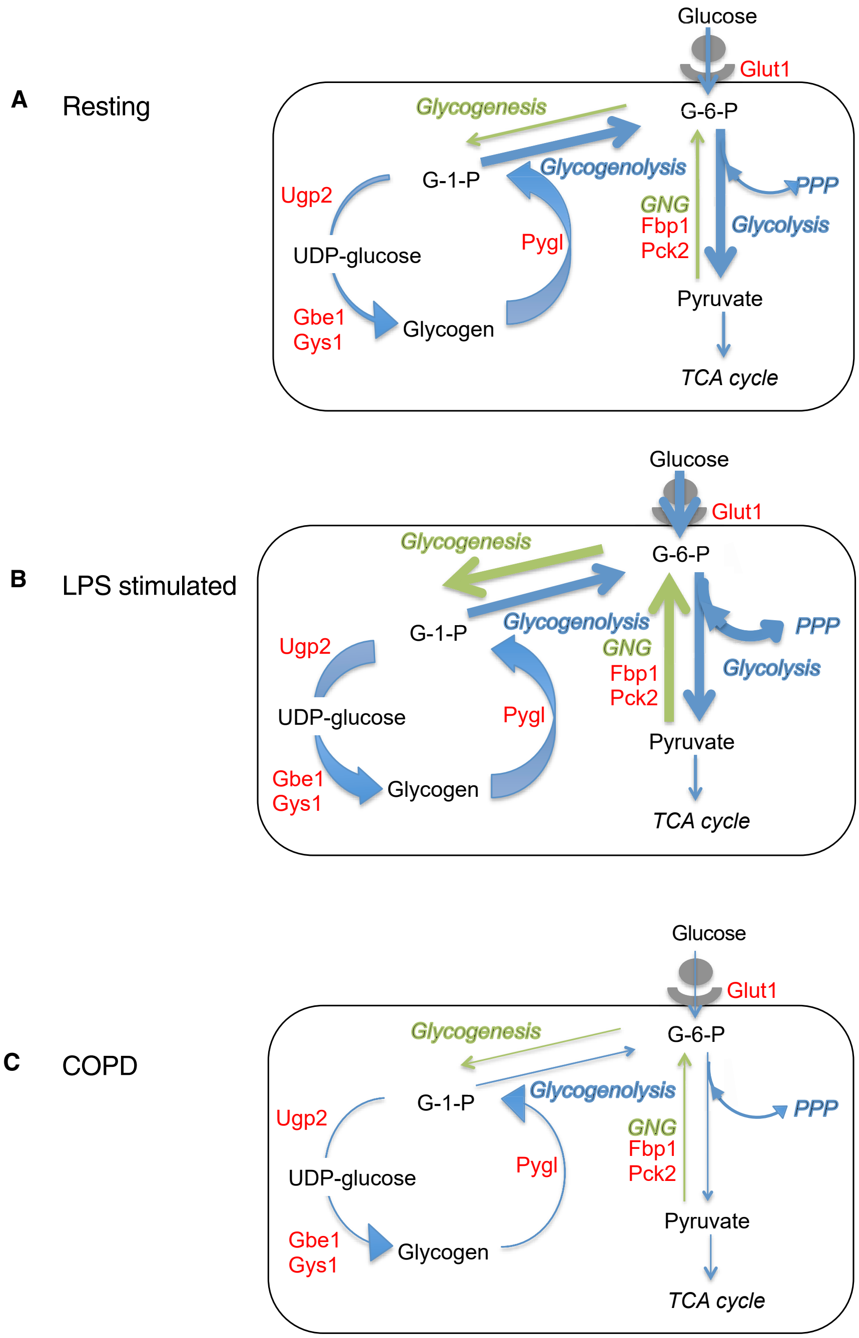

Figure 6. A Summary of the Observed Metabolic States of Quiescent, LPS Stimulated, and COPD Neutrophils

A diagram showing the metabolic states of resting (A), stimulated (B), and COPD (C) neutrophils showing increased glycolytic activity and glycogen synthesis in response to LPS and defective glycogen cycling and glycolysis in COPD. Genes identified to actively regulate neutrophil glucose transport (Glut1), gluconeogenesis (GNG) (Fbp1 and Pck2), glycogenesis (Gys1, Gbe1, and Ugp2), and glycogenolysis (Pygl) are highlighted in red. Arrow thickness indicates the relative flux through metabolic pathways with glycogenolysis and glucose oxidation highlighted in blue and glycogenesis and gluconeogenesis in green.

2012). In hepatoma cell lines, these changes in glycogen turnover are sensitive to hypoxia and important for cell survival (Pescador et al., 2010). More broadly, glycogen utilization has also been linked to cancer cell proliferation, abrogation of premature senescence (Rousset et al., 1979), and optimal functioning of the PPP (Favaro et al., 2012). The impact of hypoxia in this setting is of considerable interest given hypoxia is an important hallmark of the tumor microenvironment. This is further strengthened by the observation that glycogen biosynthetic enzymes (GYS1, GBE1, and UGP2) are themselves regulated at a transcriptional level by hypoxia (Pescador et al., 2010). In a disease setting, defective glutamine utilization for gluconeogenesis and reduced glycogen shuttling result in insufficient glycogen stores to fuel effective immune responses and generate pathogen-sensing sugars, with consequences for neutrophil survival and bacterial killing. Moreover, *in vivo* study of neutrophils deficient in the glycogen synthesis enzyme GYS1 provides direct evidence that neutrophil glycogen fuels effective host immunity, with impaired bacterial control observed in Gys1^{loxlox} MRP8-Cre$^{+/-}$ transgenic mice.

Limitations of Study

Our study demonstrates that neutrophils have adapted to use multiple metabolic substrates to generate energy stores in the

form of glycogen, which are dynamically regulated by both gluconeogenesis and glycogenesis. Increased glycogen shunting enables neutrophils to meet the higher metabolic demands of a pro-inflammatory response. Conversely, failure to replenish or access these glycogen stores results in metabolic exhaustion and dysfunctional neutrophil responses. What underpins the defective glycogen cycling we observe in neutrophils isolated from patients with chronic inflammatory disease is currently unknown, and a limitation of the work undertaken to date. Moreover, how neutrophil metabolic responses are reprogrammed and retained within circulating populations over time requires further study. Nonetheless, we propose that this metabolic specialization of neutrophils could allow selective targeting of glycogen cycling to optimize the efficacy of neutrophil host responses and thus facilitate inflammation resolution.

STAR★METHODS

Detailed methods are provided in the online version of this paper and include the following:

- KEY RESOURCES TABLE
- RESOURCE AVAILABILITY
  - Lead Contact
  - Materials Availability
  - Data and Code Availability
- EXERIMENTAL MODEL AND SUBJECT DETAILS
  - Subject Details
  - Isolation of Human Neutrophils
  - Generation of Gys1$^{lox/lox}$ MRP8-Cre+/− Mice
  - Study Approval
- METHOD DETAILS
  - Neutrophil Culture
  - Radioactive Flux Assays
  - Energy Status and Energy Charge
  - LC-MS
  - Measurement of Intracellular Glycogen Stores
  - Extracellular Acidification Rate (ECAR) Quantification Using Seahorse
  - Apoptosis Assay
  - RNA Isolation and Relative Quantification
  - Bacterial Killing Assay
  - Protein Extraction and Western Blotting
- QUANTIFICATION AND STATISTICAL ANALYSIS

SUPPLEMENTAL INFORMATION

Supplemental Information can be found online at https://doi.org/10.1016/j.cmet.2020.11.016.

ACKNOWLEDGMENTS

We thank Sandra Schoors for providing guidance and help with the radioactive assays, Wesley Vermaelen and Camila Takeno Cologna for help with LC-MS analysis, and the QMRI Flow Cytometry Facility for help with flow cytometry. This work was principally supported by Wellcome Trust Clinical Fellowship awards, UK (098516 and 209220 to S.R.W.). The work of P. Carmeliet is supported by long-term structural Methusalem funding from the Flemish Government. The Apex 5 expedition was supported by funding from the JABBS and Sylvia Waddilove Foundations, UK. Work in R.R.M.'s lab is supported by Medical Research Council (MC_PC_U127574433 and MC_UU_0007/17), UK.

AUTHOR CONTRIBUTIONS

P.S., J.A.W., B.G., P. Carmeliet, D.H.D., M.K.B.W., and S.R.W. designed the experiments. P.S., J.A.W., E.M.R., D.S., P. Coelho, E.R.W., R.G., J.M.Y., M.B., S.A., A.S.M., M.A.S.G., T.M., A.Z., L.R., T.G., P.J., G.G.P., C.J.G., J.-M.M., A.A.-S., V.M.D., G.R.-B., J.D., and J.J.G. performed the experiments. J.P.T., K.B., A.A.R.T., R.R.M., A.V.K., M.M., B.G., P. Carmeliet, M.K.B.W., and S.R.W. provided technical expertise and performed data analysis. P.S., M.K.B.W., and S.R.W. wrote the manuscript.

DECLARATION OF INTERESTS

The authors declare no competing interests.

Received: February 14, 2019
Revised: September 30, 2020
Accepted: November 20, 2020
Published: December 10, 2020; corrected online: March 24, 2021

REFERENCES

Bender, J.G., and Van Epps, D.E. (1985). Inhibition of human neutrophil function by 6-aminonicotinamide: the role of the hexose monophosphate shunt in cell activation. Immunopharmacology *10*, 191–199.

Borregaard, N., and Herlin, T. (1982). Energy metabolism of human neutrophils during phagocytosis. J. Clin. Invest. *70*, 550–557.

Boxer, L.A., Baehner, R.L., and Davis, J. (1977). The effect of 2-deoxyglucose on guinea pig polymorphonuclear leukocyte phagocytosis. J. Cell. Physiol. *91*, 89–102.

Cheung, Y.Y., Kim, S.Y., Yiu, W.H., Pan, C.-J., Jun, H.-S., Ruef, R.A., Lee, E.J., Westphal, H., Mansfield, B.C., and Chou, J.Y. (2007). Impaired neutrophil activity and increased susceptibility to bacterial infection in mice lacking glucose-6-phosphatase-β. J. Clin. Invest. *117*, 784–793.

De Bock, K., Georgiadou, M., Schoors, S., Kuchnio, A., Wong, B.W., Cantelmo, A.R., Quaegebeur, A., Ghesquière, B., Cauwenberghs, S., Eelen, G., et al. (2013). Role of PFKFB3-driven glycolysis in vessel sprouting. Cell *154*, 651–663.

Desai, H., Eschberger, K., Wrona, C., Grove, L., Agrawal, A., Grant, B., Yin, J., Parameswaran, G.I., Murphy, T., and Sethi, S. (2014). Bacterial colonization increases daily symptoms in patients with chronic obstructive pulmonary disease. Ann. Am. Thorac. Soc. *11*, 303–309.

Duran, J., Saez, I., Gruart, A., Guinovart, J.J., and Delgado-García, J.M. (2013). Impairment in long-term memory formation and learning-dependent synaptic plasticity in mice lacking glycogen synthase in the brain. J. Cereb. Blood Flow Metab. *33*, 550–556.

Favaro, E., Bensaad, K., Chong, M.G., Tennant, D.A., Ferguson, D.J.P., Snell, C., Steers, G., Turley, H., Li, J.L., Günther, U.L., et al. (2012). Glucose utilization via glycogen phosphorylase sustains proliferation and prevents premature senescence in cancer cells. Cell Metab. *16*, 751–764.

Finisguerra, V., Di Conza, G., Di Matteo, M., Serneels, J., Costa, S., Thompson, A.A.R., Wauters, E., Walmsley, S., Prenen, H., Granot, Z., et al. (2015). MET is required for the recruitment of anti-tumoural neutrophils. Nature *522*, 349–353.

Fossati, G., Moulding, D.A., Spiller, D.G., Moots, R.J., White, M.R.H., and Edwards, S.W. (2003). The mitochondrial network of human neutrophils: role in chemotaxis, phagocytosis, respiratory burst activation, and commitment to apoptosis. J. Immunol. *170*, 1964–1972.

Garnett, J.P., Nguyen, T.T., Moffatt, J.D., Pelham, E.R., Kalsi, K.K., Baker, E.H., and Baines, D.L. (2012). Proinflammatory mediators disrupt glucose homeostasis in airway surface liquid. J. Immunol. *189*, 373–380.

Hogg, J.C., Chu, F., Utokaparch, S., Woods, R., Elliott, W.M., Buzatu, L., Cherniack, R.M., Rogers, R.M., Sciurba, F.C., Coxson, H.O., and Paré, P.D. (2004). The nature of small-airway obstruction in chronic obstructive pulmonary disease. N. Engl. J. Med. *350*, 2645–2653.

Jha, A.K., Huang, S.C.C., Sergushichev, A., Lampropoulou, V., Ivanova, Y., Loginicheva, E., Chmielewski, K., Stewart, K.M., Ashall, J., Everts, B., et al. (2015). Network integration of parallel metabolic and transcriptional data

reveals metabolic modules that regulate macrophage polarization. Immunity *42*, 419–430.

Jun, H.S., Cheung, Y.Y., Lee, Y.M., Mansfield, B.C., and Chou, J.Y. (2012). Glucose-6-phosphatase-β, implicated in a congenital neutropenia syndrome, is essential for macrophage energy homeostasis and functionality. Blood *119*, 4047–4055.

Kim, S.Y., Jun, H.S., Mead, P.A., Mansfield, B.C., and Chou, J.Y. (2008). Neutrophil stress and apoptosis underlie myeloid dysfunction in glycogen storage disease type Ib. Blood *111*, 5704–5711.

Ko, C.W., Counihan, D., Wu, J., Hatzoglou, M., Puchowicz, M.A., and Croniger, C.M. (2018). Macrophages with a deletion of the *phosphoenolpyruvate carboxykinase 1* (*Pck1*) gene have a more proinflammatory phenotype. J. Biol. Chem. *293*, 3399–3409.

Ma, R., Ji, T., Zhang, H., Dong, W., Chen, X., Xu, P., Chen, D., Liang, X., Yin, X., Liu, Y., et al. (2018). A Pck1-directed glycogen metabolic program regulates formation and maintenance of memory $CD8^+$ T cells. Nat. Cell Biol. *20*, 21–27.

Martin, W.H., Hoover, D.J., Armento, S.J., Stock, I.A., McPherson, R.K., Danley, D.E., Stevenson, R.W., Barrett, E.J., and Treadway, J.L. (1998). Discovery of a human liver glycogen phosphorylase inhibitor that lowers blood glucose in vivo. Proc. Natl. Acad. Sci. USA *95*, 1776–1781.

Mills, E.L., Kelly, B., Logan, A., Costa, A.S.H., Varma, M., Bryant, C.E., Tourlomousis, P., Däbritz, J.H.M., Gottlieb, E., Latorre, I., et al. (2016). Succinate dehydrogenase supports metabolic repurposing of mitochondria to drive inflammatory macrophages. Cell *167*, 457–470.e13.

Naughton, D., Whelan, M., Smith, E.C., Williams, R., Blake, D.R., and Grootveld, M. (1993). An investigation of the abnormal metabolic status of synovial fluid from patients with rheumatoid arthritis by high field proton nuclear magnetic resonance spectroscopy. FEBS Lett. *317*, 135–138.

Neubert, D., and Lehninger, A.L. (1962). The effect of oligomycin, gramicidin and other antibiotics on reversal of mitochondrial swelling by adenosine triphosphate. Biochim. Biophys. Acta *62*, 556–565.

Obel, L.F., Müller, M.S., Walls, A.B., Sickmann, H.M., Bak, L.K., Waagepetersen, H.S., and Schousboe, A. (2012). Brain glycogen-new perspectives on its metabolic function and regulation at the subcellular level. Front. Neuroenergetics *4*, 3.

Pescador, N., Villar, D., Cifuentes, D., Garcia-Rocha, M., Ortiz-Barahona, A., Vazquez, S., Ordoñez, A., Cuevas, Y., Saez-Morales, D., Garcia-Bermejo, M.L., et al. (2010). Hypoxia promotes glycogen accumulation through hypoxia inducible factor (HIF)-mediated induction of glycogen synthase 1. PLoS ONE *5*, e9644.

Pike, L.S., Smift, A.L., Croteau, N.J., Ferrick, D.A., and Wu, M. (2011). Inhibition of fatty acid oxidation by etomoxir impairs NADPH production and increases reactive oxygen species resulting in ATP depletion and cell death in human glioblastoma cells. Biochim. Biophys. Acta *1807*, 726–734.

Raud, B., Roy, D.G., Divakaruni, A.S., Tarasenko, T.N., Franke, R., Ma, E.H., Samborska, B., Hsieh, W.Y., Wong, A.H., Stüve, P., et al. (2018). Etomoxir actions on regulatory and memory T cells are independent of Cpt1a-mediated fatty acid oxidation. Cell Metab. *28*, 504–515.e7.

Robinson, J.M., Karnovsky, M.L., and Karnovsky, M.J. (1982). Glycogen accumulation in polymorphonuclear leukocytes, and other intracellular alterations that occur during inflammation. J. Cell Biol. *95*, 933–942.

Rousset, M., Chevalier, G., Rousset, J.P., Dussaulx, E., and Zweibaum, A. (1979). Presence and cell growth-related variations of glycogen in human colorectal adenocarcinoma cell lines in culture. Cancer Res. *39*, 531–534.

Sadiku, P., Willson, J.A., Dickinson, R.S., Murphy, F., Harris, A.J., Lewis, A., Sammut, D., Mirchandani, A.S., Ryan, E., Watts, E.R., et al. (2017). Prolyl hydroxylase 2 inactivation enhances glycogen storage and promotes excessive neutrophilic responses. J. Clin. Invest. *127*, 3407–3420.

Sbarra, A.J., and Karnovsky, M.L. (1959). The biochemical basis of phagocytosis. I. Metabolic changes during the ingestion of particles by polymorphonuclear leukocytes. J. Biol. Chem. *234*, 1355–1362.

Scott, R.B. (1968). Glycogen in human peripheral blood leukocytes. I. Characteristics of the synthesis and turnover of glycogen in vitro. J. Clin. Invest. *47*, 344–352.

Thompson, A.A.R., Dickinson, R.S., Murphy, F., Thomson, J.P., Marriott, H.M., Tavares, A., Willson, J., Williams, L., Lewis, A., Mirchandani, A., et al. (2017). Hypoxia determines survival outcomes of bacterial infection through HIF-1α dependent re-programming of leukocyte metabolism. Sci. Immunol. *2*, eaal2861.

Thwe, P.M., Pelgrom, L.R., Cooper, R., Beauchamp, S., Reisz, J.A., D'Alessandro, A., Everts, B., and Amiel, E. (2017). Cell-intrinsic glycogen metabolism supports early glycolytic reprogramming required for dendritic cell immune responses. Cell Metab. *26*, 558–567.e5.

Tyrakis, P.A., Palazon, A., Macias, D., Lee, K.L., Phan, A.T., Veliça, P., You, J., Chia, G.S., Sim, J., Doedens, A., et al. (2016). S-2-hydroxyglutarate regulates $CD8^+$ T-lymphocyte fate. Nature *540*, 236–241.

Valentine, W.N., Follette, J.H., and Lawrence, J.S. (1953). The glycogen content of human leukocytes in health and in various disease states. J. Clin. Invest. *32*, 251–257.

Vincent, E.E., Sergushichev, A., Griss, T., Gingras, M.C., Samborska, B., Ntimbane, T., Coelho, P.P., Blagih, J., Raissi, T.C., Choinière, L., et al. (2015). Mitochondrial phosphoenolpyruvate carboxykinase regulates metabolic adaptation and enables glucose-independent tumor growth. Mol. Cell *60*, 195–207.

Walmsley, S.R., Print, C., Farahi, N., Peyssonnaux, C., Johnson, R.S., Cramer, T., Sobolewski, A., Condliffe, A.M., Cowburn, A.S., Johnson, N., and Chilvers, E.R. (2005). Hypoxia-induced neutrophil survival is mediated by HIF-1α-dependent NF-kappaB activity. J. Exp. Med. *201*, 105–115.

Wick, A.N., Drury, D.R., Nakada, H.I., and Wolfe, J.B. (1957). Localization of the primary metabolic block produced by 2-deoxyglucose. J. Biol. Chem. *224*, 963–969.

Yao, C.H., Liu, G.Y., Wang, R., Moon, S.H., Gross, R.W., and Patti, G.J. (2018). Identifying off-target effects of etomoxir reveals that carnitine palmitoyltransferase I is essential for cancer cell proliferation independent of β-oxidation. PLoS Biol. *16*, e2003782.

Yu, S.M., and Kim, S.J. (2010). Endoplasmic reticulum stress (ER-stress) by 2-deoxy-D-glucose (2DG) reduces cyclooxygenase-2 (COX-2) expression and N-glycosylation and induces a loss of COX-2 activity via a Src kinase-dependent pathway in rabbit articular chondrocytes. Exp. Mol. Med. *42*, 777–786.

STAR★METHODS

KEY RESOURCES TABLE

REAGENT or RESOURCE	SOURCE	IDENTIFIER
Antibodies		
Anti-PCK2 antibody	Abcam	Cat# ab70359; RRID: AB_1952317
Anti-FBP1 antibody	Abcam	Cat# ab109020; RRID: AB_10865049
Anti-p38 MAPK Antibody	New England Biolabs	Cat# 9212S; RRID: AB_330713
Anti-Phospho-Glycogen Synthase (Ser641) Antibody	Cell Signaling	Cat# 3891S; RRID: AB_2116390
Anti-PYGL antibody	Abcam	Cat# ab198268-100ul
Bacterial and Virus Strains		
Staphyloccus aureus SH1000	Prof Simon Foster	N/A
Streptococcus pneumoniae S14	Prof David Dockrell	N/A
Biological Samples		
Healthy adult blood neutrophils	Isolated from healthy volunteers	N/A
COPD adult blood neutrophils	Isolated from COPD patients	N/A
Chemicals, Peptides, and Recombinant Proteins		
Fructose-1,6-bisphosphatase-1 Inhibitor	Cambridge BioScience	Cat#18860-5mg-CAY
GLUTAMINASE INHIBITOR II BPTES	Merck Life Sciences UK	Cat#5300300001
AMYLOGLUCOSIDASE FROM ASPERGILLUS NIGER LYOPH. ~70 U/MG	Merck Life Sciences UK	Cat#10115-1G-F
CP-91149	Sigma-Aldrich	Cat#PZ0104-5M
3-Mercaptopicolinic acid (HCl)	Cambridge bioscience	Cat#20895-100 mg-CAY
MB05032 (Tocris Bioscience)	Bio-Techne	Cat#6618/10
Etomoxir	Abcam	Cat#ab144763-2mg
LPS from *E. coli*, Serotype R515 (Re) (TLRgrade)	Enzo life Sciences Europe	Cat#ALX-581-007-L002
Critical Commercial Assays		
EasySep Human neutrophil Isolation Kit	Scientific Laboratory Supplies Ltd	Cat#17957
Glycogen assay kit	Sigma-Aldrich	Cat#MAK016
FBP1, Homo_sapiens PrimeTime Std qPCR Assay	Integrated DNA Technologies	Cat#Hs.PT.58.1719755
GYS1, Homo_sapiens PrimeTime Std qPCR Assay	Integrated DNA Technologies	N/A
ACTB, Homo_sapiens PrimeTime Std qPCR Assay	Integrated DNA Technologies	Cat#Hs.PT.39a.22214847
PYGL (phosphorylase, glycogen, liver), Hs00958087_m1	Applied Biosystems	Cat#4331182
SLC2A1 Homo_sapiens PrimeTime Std qPCR Assay	Integrated DNA Technologies	Cat#Hs.PT.58.25872862
PCK2 Homo_sapiens PrimeTime Std qPCR Assay	Integrated DNA Technologies	Cat#Hs.PT.58.19439369
UGP2 Homo_sapiens PrimeTime Std qPCR Assay	Integrated DNA Technologies	Cat#Hs.58.39665284
Seahorse XF Glycolytic Rate Assay	Agilent Technologies	Cat#Cat#103344-100
Experimental Models: Organisms/Strains		
$Gys1l^{ox/lox}$ MRP8-$Cre^{+/-}$ mice	In this paper	N/A
C57BL/6JOlaHsd	Jackson Laboratory	N/A
Software and Algorithms		
GraphPad Prism v.8	GraphPad Software	GraphPad Prism; RRID: SCR_002798
Thermo Xcalibur	Thermo Fisher Scientific	Thermo Fisher Scientific

RESOURCE AVAILABILITY

Lead Contact

Further information and requests for resources and reagents should be directed to and will be fulfilled by the Lead Contact, sarah.walmsley@ed.ac.uk

CellPress
OPEN ACCESS

Materials Availability

The mouse line (Gys1$^{lox/lox}$ MRP8-Cre+/−) generated in this study is available from the Lead Contact upon request.

Data and Code Availability

This study did not generate/analyze datasets/code.

EXERIMENTAL MODEL AND SUBJECT DETAILS

Subject Details

Peripheral venous blood neutrophils were isolated from healthy volunteers with written informed consent as approved by the CIR Blood Resource Management Committee (AMREC 15-HV-013) in Edinburgh and the Commisie Medische Ethiek UZ KU Leuven in Belgium (total number of participants 67 aged between 20-70 years, mean age range 31-40, Gender M 27: F 40). Peripheral venous blood neutrophils were also isolated from patients with COPD in accordance with local ethics (REC reference: 15/SS/0095). Patient details are shown in Table S1 (total number of participants 24 aged 40-76 years, mean age 58.7, Gender M 10: F 14). Exclusion criteria included history of anemia, pregnancy or breast-feeding, Diabetes Mellitus, CKD, hepatic failure, immunosuppression and febrile illness within the last two weeks. COPD patients were matched to healthy non-smoker control donors ± 5 years of age wherever possible (total number of participants 18 aged between 20-70, mean age range 41-50, Gender M 8: F 9). Negative magnetic selection (STEMCELL Technologies, Vancouver) was carried out to further purify the neutrophils and consistently demonstrated purity of over 98%.

High Altitude Research Participants

12 healthy participants aged 19-22 years (mean age 20, Gender M 6:F 6) were recruited from the University of Edinburgh student community. Exclusion criteria included previous hospital admission for asthma, significant cardio-respiratory disease, regular cardiovascular medications, pregnancy, smoking and previous exposure to high altitude (defined as > 2500 m). Ethical approval was granted by the ACCORD Medical Research Ethics Committee (REC reference: 17-HV-030) and all participants provided informed written consent. Data were collected at sea level, during baseline and post-expedition testing, and at 4,700 m on the APEX 5 expedition. Subjects flew into La Paz, Bolivia (3,600 m) within a 12 h period and after acclimatizing for four days, ascended 90 min by bus to Huayna Potosí base camp (4,700 m) for seven days before returning to La Paz. Mean peripheral blood oxygen saturation (SpO_2) was measured daily using pulse oximetry (Santa Medical, Tustin). In all control and patient cohorts both male and female participants were included and matched where possible, small sample sizes precluded any further study of the influence of sex.

Isolation of Human Neutrophils

Neutrophils were isolated from sodium citrate anticoagulated blood as previously described using dextran sedimentation and discontinuous percoll gradients (Walmsley et al., 2005).

Generation of Gys1$^{lox/lox}$ MRP8-Cre+/− Mice

Gys1 $^{lox/lox}$ mice (Duran et al., 2013) were crossed with the previously reported neutrophil specific cre driver Mrp8 (Finisguerra et al., 2015) to generate Gys1$^{lox/lox}$ MRP8-Cre+/− mice on a C57BL/6JOla genetic background. Both male and female littermates were used from ages 6-12 weeks old with Gys1$^{lox/lox}$ MRP8-Cre−/− mice and wildtype C57BL/6JOla sex matched or litter mate controls.

Study Approval

Animal experiments were conducted in accordance with the UK Home Office Animals (Scientific Procedures) Act of 1986. All animal studies were approved by The University of Edinburgh Animal Welfare and Ethical Review Board. 6-12 week old male and female mice were used for experiments. Mice were housed in IVC cages under 12 h light/darkness cycles and controlled temperature (20-23°C) in accordance with UK Home Office guidance. All mice used for experiments were healthy with quarterly and annual testing carried out in accordance with FELASA 2014 Guidelines, using a mixture of environmental, random colony samples and sentinel testing by serology and PCR. Mice had free access to food (Special diets service rat and mouse number 1 maintenance food RMI (P) 801151) and water.

METHOD DETAILS

Neutrophil Culture

Neutrophils were cultured at 5 million cells/mL for 2-20 h at 37°C, in normoxia (21% O2, 5% CO2) or hypoxia (1% (3kPa) O2, 5% CO2) in two types of pre-equilibrated RPMI media 1640 with 10% fetal bovine serum (FBS) (GIBCO) and 1% Penicillin/Streptomycin, differing in the presence or absence of glucose. In the absence of glucose, dialyzed glucose-free FBS was used. To study the effects of stimulation and metabolic inhibitors, neutrophils were cultured with (100ng/mL) LPS *E.coli* serotype R515 (Enzo), CP-91149 (Sigma-Aldrich), 2-deoxyglucose (Sigma-Aldrich), Oligomycin A (Sigma-Aldrich) and Etomoxir (10uM) (CNIO Carlos III Therapies) (Raud et al., 2018; Yao et al., 2018), Fructose-1,6-bisphosphatase-1 Inhibitor (FBP1i) (Cambridge Bioscience), MB05032 (Tocris Bioscience), 3-Mercaptopicolinic acid (HCl) (3-MPA) (Cambridge Bioscience), Glutaminase Inhibitor II, BPTES (MERCK Chemical).

Radioactive Flux Assays

^{3}H-Glycolytic Flux

Purified peripheral blood neutrophils (2.5 x10^6) were incubated for 6 h in RPMI 1640 medium (supplemented with 5.5 mM unlabeled glucose, 10% FCS and 1% Penicillin/Streptomycin) containing 0.4 μCi/mL [5-^{3}H]-D-glucose (Perkin Elmer). Cells were pelleted (420 g for 10 min) and supernatant transferred into glass vials containing 12% perchloric acid sealed with rubber stoppers. ^3H_2O was captured in hanging wells containing a piece of Whatman paper soaked with H_2O over a period of 48 h at 37°C to reach saturation. Radioactivity in the Whatman paper was determined by liquid scintillation counting.

^{3}H- Fatty Acid Flux

2.5 x10^6 were incubated 6 h in RPMI 1640 medium (supplemented with 5.5 mM unlabeled glucose, 10% FCS and 1% Penicillin/Streptomycin) containing 0.4 μCi/mL [-^{3}H]-D-palmitic acid (Perkin Elmer).

^{14}C-glucose Oxidation

The contribution of the TCA cycle to total glucose utilization was measured as $^{14}CO_2$ release formation both in the TCA cycle and oxidative pentose phosphate pathway using 0.55 μCi/mL [6- ^{14}C]-D-glucose. Following a 6 h incubation in RPMI 1640 medium (supplemented with 5.5 mM unlabeled glucose, 10% FCS and 1% Penicillin/Streptomycin) containing 0.55 μCi/mL [6- ^{14}C]-D-glucose, 2.5 x10^6 cells were lysed using 250 μL of 12% Perchloric acid. Wells were immediately covered with a piece of 1x hyamine hydroxide-saturated Whatman paper and overnight absorption of $^{14}CO_2$ released was performed at room temperature. Radioactivity in the Whatman paper was measured using scintillation counting.

Energy Status and Energy Charge

A total of 5x10^6 purified human peripheral blood neutrophils were harvested in 100 μL of ice-cold 5% PCA supplemented with 1 mM EDTA. ATP, ADP and AMP levels were measured using ion-pair RP-HLPC. Thereafter, samples were centrifuged for 10 min at 13000 g and the supernatant neutralized with $K2CO_3$. Following perchlorate precipitation, the supernatant was injected on a 4.6 × 150-mm, 5-μm particle size C-18 HPLC column at a rate of 1 mL/min, 100% buffer A from 0 to 5 min, 100% buffer A to 100% buffer B from 5 to 20 min, 100% buffer B from 20 to 31 min for column re-equilibration (buffer A: 25 mM NaH2PO4, 0.385 mM tetrabutylammonium, pH 5; buffer B: 10% (v/v) acetonitrile in 200 mM NaH2PO4, 0.385 mM tetrabutylammonium, pH 4). Phosphorylated nucleotides were monitored at 260 nm. The energy charge was expressed as ([ATP] + 1/2 [ADP] / [ATP] + [ADP] +[-AMP]) and the energy status of the cells as the ratio of ATP to ADP content.

LC-MS

Ultrapure neutrophils were cultured in the presence of 5.5 mM. U-^{13}C glucose or 100 μM U-^{13}C palmitate for 4 h and 2mM U-^{13}C glutamine for 2,4 and 6 h. 2 0.5x10^6 cells were harvested in 100 μL of 80% methanol. Measurements of relative levels of analyte abundance and ^{13}C incorporation into glycolytic intermediates were performed using a Dionex UltiMate 3000 LC System (Thermo Scientific) coupled to a Q Exactive Orbitrap Mass Spectrometer (Thermo Scientific) operated in negative mode. Practically, 25 μL of sample was injected on a SeQuant ZIC/pHILIC Polymeric column (Merck Millipore). The gradient started with 10% solvent B (10 mM NH4-acetate in mqH2O, pH 9.3) and 90% solvent A (acetonitrile) and remained at 10% B until 2 min after injection. Next, a linear gradient to 80% B was carried out until 29 min. At 38 min, the gradient returned to 40% B, followed by a decrease to 10% B at 42 min. The chromatography was stopped at 58 min. The flow was kept constant at 100 μl/min, and the column was kept at 25°C throughout the analysis. The MS operated in full scan–SIM mode using a spray voltage of 3.2 kV, capillary temperature of 320°C, sheath gas at 10.0, auxiliary gas at 5.0. AGC target was set at 1e6 using a resolution of 140,000, with a maximum IT of 500 ms. Data collection and analysis were performed using Xcalibur Software (Thermo Scientific). Total metabolite abundance is shown as a measure of both unlabeled ^{12}C isotopologues and all other mass isotopologues corresponding to the analyte of interest. Isotope correction was carried out as previously described using an in-house software tool.

LC-MS Analysis of Glucose Generated from Hydrolysed Glycogen

Neutrophils (10 x10^6) were cultured in U-^{13}C pyruvate for 8 h under normoxia and hypoxia. The cells were washed with saline and lysed by boiling for 15 min in 30% KOH. Glycogen was precipitated using ice cold 100% ethanol and washed a further two times. Following the evaporation of residual ethanol and resuspension in water the glycogen was hydrolysed using amyloglucosidase (Merck Life Sciences UK). Acetonitrile:methanol:water 40:40:20 was added to the samples and samples analyzed by LC-MS.

Measurement of Intracellular Glycogen Stores

Radioactive Assays for Measuring Glycogen Content

Neutrophils (5 × 10^6) were culture with U-^{14}C glucose or U-^{14}C lactate for 6 h in the absence and presence of LPS under normoxia and hypoxia. The cells were washed 5 times in ice-cold PBS and lysed in 30% KOH by boiling for 15 min. An aliquot of the homogenate was spotted onto Whatman paper and the glycogen precipitated by immersing the paper in ice-cold 66% ethanol (v/v). Following two washes with ethanol, radioactivity in the Whatman paper was measured using scintillation counting.

Fluorimetric Assay

1 × 10^6 human neutrophils were lysed in 200 μL ice-cold H2O and boiled for 10 min at 95 degrees; lysates were centrifuged at 18,000 g at 4°C for 10 min to remove cell debris and snap frozen. Glycogen content was measure using a fluorimetric assay (Sigma-Aldrich).

Extracellular Acidification Rate (ECAR) Quantification Using Seahorse

Neutrophils were resuspended in XF assay media at a concentration of $3x10^6$/mL onto a XF24 cell plate pre-coated with Cell-Tak (Corning). Cells were stimulated with *Staphylococcus aureus* (SH1000) at MOI 10, 25 and 50. The ECAR were measured at intervals of 7 min over a 90-min cycle using a Seahorse XF24 (Seahorse Bioscience USA).

Apoptosis Assay

Neutrophil apoptosis following 20 h of culture was measured by flow cytometry of cell stained with Annexin V PE and the nuclear dye TO-PRO-3 (Molecular Probes, Thermo Fisher). Flow cytometric measurements were performed using a four-color FACSCalibur flow cytometer (Becton Dickinson).

RNA Isolation and Relative Quantification

RNA was isolated from neutrophils using the mirVana total RNA isolation protocol (Ambion, Thermo Fisher), DNase treated and reverse transcribed using AMV reverse transcriptase with random primers (Promega). TaqMan gene expression assays (Applied Biosystems, Thermo Fisher) and PrimeTime qPCR Probe Assays (IDT) were used for relative quantification of cDNA using SDS 2.4 (Thermo Fisher) and normalized to *ACTB* expression.

Bacterial Killing Assay

Neutrophils were infected for 90 min with an MOI of 10. After the killing of extraceullar bacteria for 30 min with either Gentamycin (20 μg/mL) and Benzylpenicillin (40 units/mL) when using serotype 14 Streptococcus pneumoniae (S14) or Gentamycin (20 μg/mL) and Vancomycin (10 μM) when using *Staphylococcus aureus* (SH1000), the numbers of internalised bacteria at times 0 and 3 h were measured.

Inhibition of Glutaminolysis (BPTES) and Gluconeogenesis (3-MPA, FBPi) and Bacterial Killing Assay

Neutrophils were cultured for 4 h in glucose-deprived media with the inhibitors and infected with *Staphylococcus aureus* SH1000 for 60 min with an MOI of 10. After the killing of extraceullar bacteria for 30 min with either Gentamycin (20 μg/mL) and Vancomycin (10 μM), the number of internalised bacteria at times 0 and 2 h were measured.

Protein Extraction and Western Blotting

Neutrophil whole cell lysates were prepared with Laemmli buffer containing PI cocktail (protease inhibitor), PhosSTOP, PMSF (phenylmethylsulfonylfluoride) and DFP (Diisopropyl fluorophosphate). Following lysis samples were boiled with 2x loading buffer containing SDS. Immunoblotting was performed with antibodies against glycogen synthase (total), phosphoglycogen synthase (Ser641) (Cell Signaling), glycogen phosphorylase (liver form) (Abcam), phosphoenol pyruvate carboxykinase 2 (PCK2) (Abcam), fructose 1,6 bisphosphatase 1 (FBP1) (Abcam) and HRP-conjugated secondaries: anti-mouse IgG (Cell Signaling Technology) and goat anti-rabbit IgG (Dako). Total P38 was used as a loading control.

In Vivo Staphylococcus aureus *Skin Abscess Model*

Mice were injected subcutaneously with live stationary phase *Staphylococcus aureus* (SH1000) at $5x10^7$ CFU. At 24 h, rectal temperatures and abscess areas were recorded, mice anesthetized and exsanguinated, and tissue processed for CFU counts.

QUANTIFICATION AND STATISTICAL ANALYSIS

Data represent mean ± SEM from at least three independent experiments.

Significance was determined by paired 2-tailed t tests unless otherwise stated. All statistical tests were performed with Prism 7 software (GraphPad Software) using unpaired t tests, paired t tests, one-way or two-way ANOVA with Tukey's multiple comparisons test, two-way ANOVA with Sidak's multiple comparions test. Statistical parametric analyses were performed following confirmation of normal distribution of the data. $^*p < 0.05$ was considered as statistically significant. $^{**}p < 0.01$, $^{***}p < 0.005$, $^{****}p < 0.001$. Statistical parameters are highlighted by * values in each figure with details provided in the associated figure legends.

Cell Metabolism, Volume *33*

Supplemental information

Neutrophils Fuel Effective Immune Responses through Gluconeogenesis and Glycogenesis

Pranvera Sadiku, Joseph A. Willson, Eilise M. Ryan, David Sammut, Patricia Coelho, Emily R. Watts, Robert Grecian, Jason M. Young, Martin Bewley, Simone Arienti, Ananda S. Mirchandani, Manuel A. Sanchez Garcia, Tyler Morrison, Ailing Zhang, Leila Reyes, Tobias Griessler, Privjyot Jheeta, Gordon G. Paterson, Christopher J. Graham, John P. Thomson, Kenneth Baillie, A.A. Roger Thompson, Jessie-May Morgan, Abel Acosta-Sanchez, Veronica M. Dardé, Jordi Duran, Joan J. Guinovart, Gio Rodriguez-Blanco, Alex Von Kriegsheim, Richard R. Meehan, Massimiliano Mazzone, David H. Dockrell, Bart Ghesquiere, Peter Carmeliet, Moira K.B. Whyte, and Sarah R. Walmsley

Supplemental Data

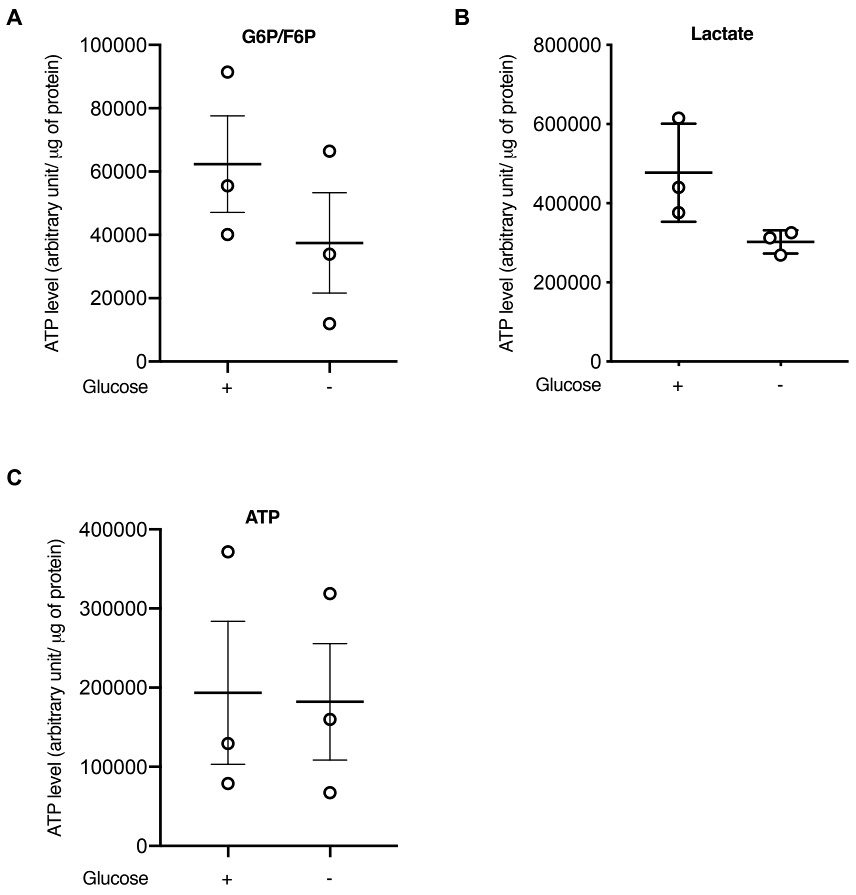

Figure S1. Total abundance of glucose-6-phosphate, lactate and ATP normalised to the protein content. Related to figure 2

Total abundance of glucose-6-phosphate (A), lactate (B) and ATP (C) normalised to the protein content are shown. Data represent mean ±SEM, n=3.

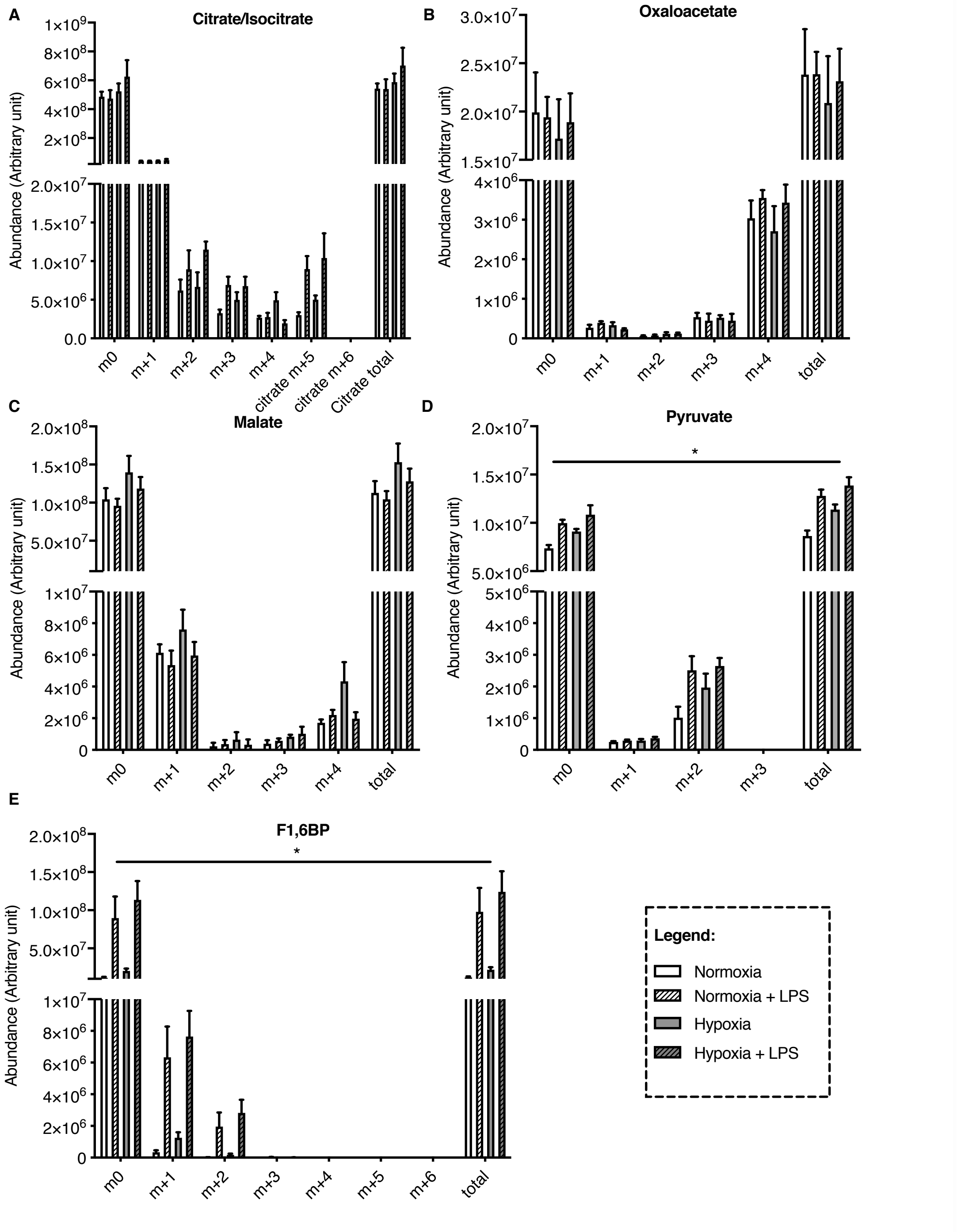

Figure S2. TCA intermediary isotopologue profiles following 4 hours of U-^{13}C glutamine culture. Related to figure 3

Neutrophils were cultured with U-^{13}C glutamine for 4 h in the presence and absence of LPS under normoxia and hypoxia. Full isotopologue profiles generated through U-^{13}C glutamine utilization are shown for citrate/isocitrate (A), oxaloacetate (B), malate (C), pyruvate (D) and fructose-1,6-bisphosphate (F1,6BP) (E). Data representative of mean ± SEM, n=4. Statistical significance was determined by 2-way ANOVA with Tukey's multiple comparisons test. *P<0.05, ****P<0.0001.

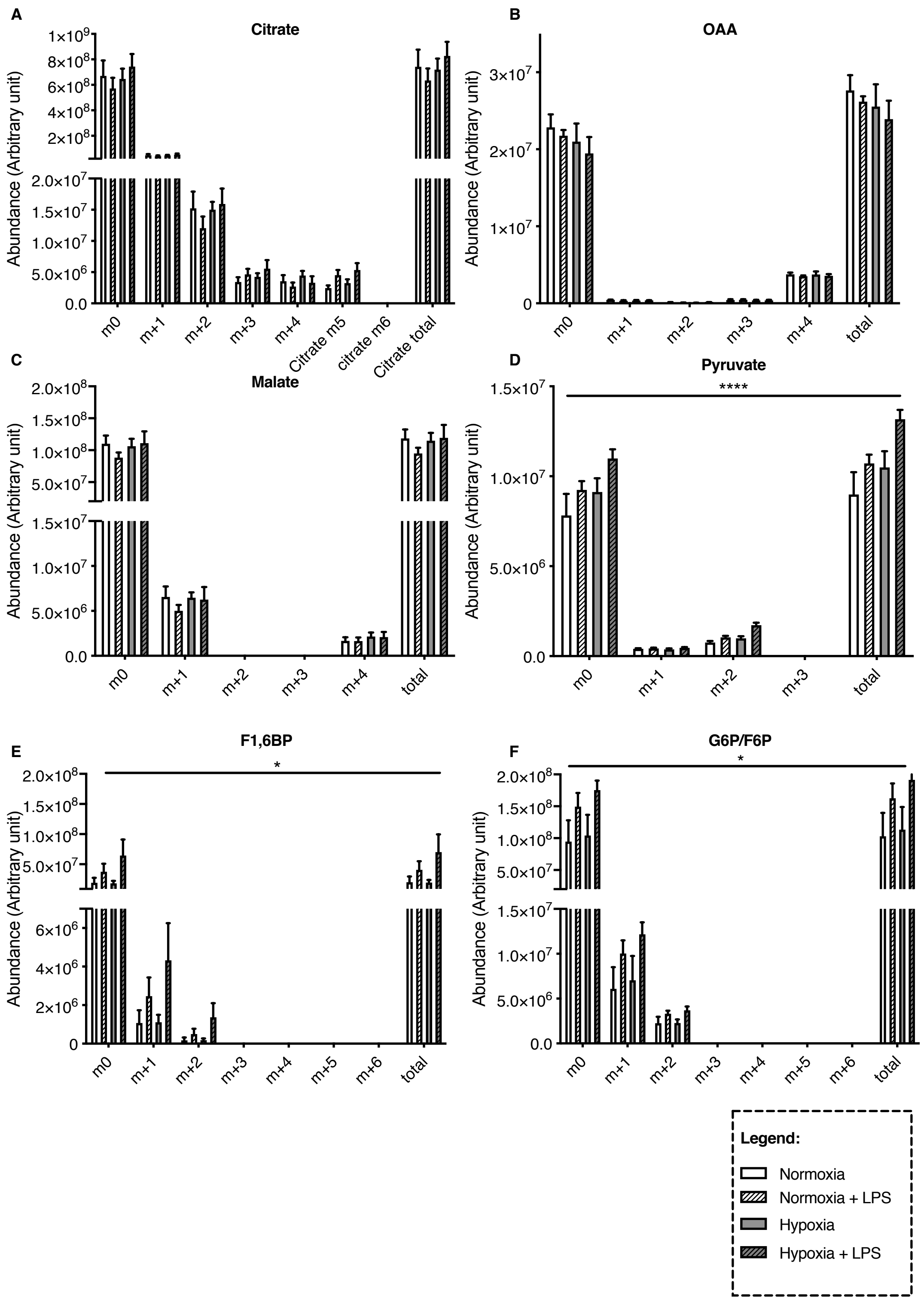

Figure S3. TCA intermediary isotopologue profiles following 2 hours of U-^{13}C glutamine culture. Related to figure 3

Neutrophils were cultured with U-^{13}C glutamine for 2 h in the presence and absence of LPS under normoxia and hypoxia. Full isotopologue profiles generated through U-^{13}C glutamine utilization are shown for citrate/isocitrate (A), oxaloacetate (B), malate (C), pyruvate (D), fructose-1,6-bisphosphate (F1,6BP) (E) and glucose-6-phosphate/fructose-6-phosphate (F). Data representative of mean ± SEM, n=4. Statistical significance was determined by 2-way ANOVA with Tukey's multiple comparisons test. *$P<0.05$, ****$P<0.0001$.

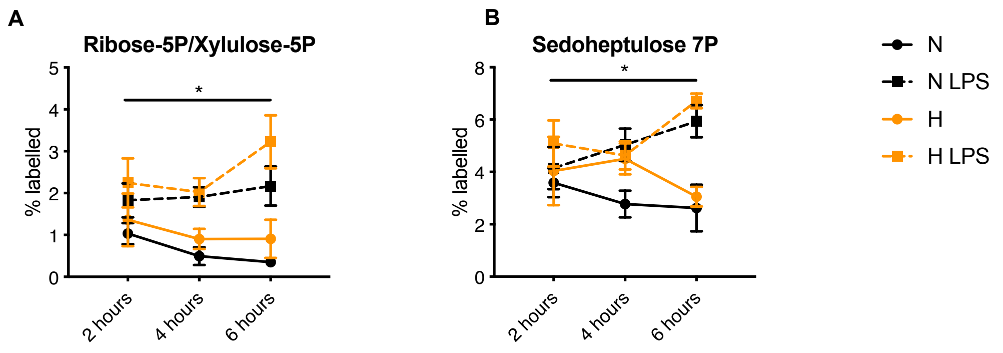

Figure S4. % labelling of PPP metabolites following 6 hours of U-^{13}C glutamine culture. Related to figure 3

Neutrophils were cultured in the presence of 13C-Glutamine for 2 (n=4), 4 (n=4) and 6 h (n=3). LC-MS time-course analyses of neutrophil % labelling of PPP metabolites Ribose-5 phosphate/xylulose-5 phosphate and Seduheptulose-7 phosphate, Data represent mean ±SEM. P values obtained via 2-way ANOVA.

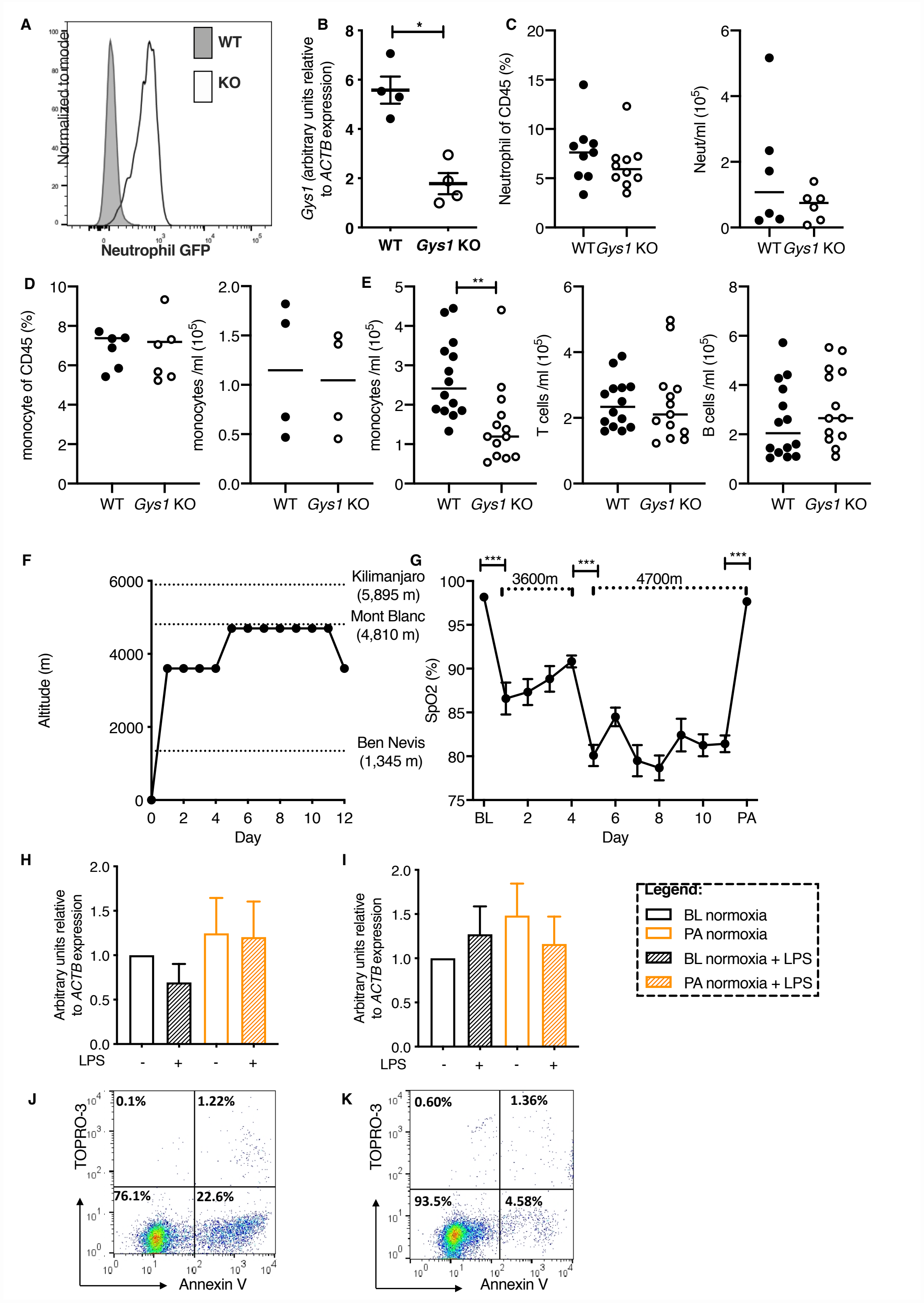

Figure S5. Gys1 deficiency and altitude induced systemic hypoxia regulate circulating blood leukocyte numbers and survival in mice and humans. Related to figure 4

Representative histogram of neutrophil expression of MRP8 driven GFP by flow cytometry comparing *Gys1* $^{lox/lox}$ MRP8-Cre$^{+/-}$ knockout (*GYS1* KO) mice, with *Gys1* $^{lox/lox}$ MRP8-Cre$^{-/-}$ wildtype (WT) (A). Quantitative PCR analysis of *Gys1* transcript expression in WT and Gys1 knockout mice (B), n=4. Proportion and number of circulating neutrophils (C) and monocytes (D) in WT and *GYS1*KO mice at baseline. Blood monocyte, T cell and B cell numbers in WT and KO mice 24 hours post *Staphylococcus aureus* infection (E). Graphical representation of subject exposure to high altitude following travel into La Paz, Bolivia (3,600m) and ascension to Huayna Potosí base camp (4,700m) (F). Mean peripheral blood oxygen saturation SpO2 was measured using pulse oximetry at baseline (BL), daily at high altitude, and three-months post-altitude (PA) (G), n = 12. Transcript expression of GBE1 (H), n=8 and GLUT1 (I), n=7 in peripheral blood neutrophils at BL and 3 months PA following 4 h of culture in the presence and absence of LPS under normoxia. Representative flow cytometry plots of Annexin V/TO-PRO-3 staining of BL (J) and 3 months PA (K) peripheral blood neutrophils following stimulation with LPS for 20 h under hypoxia. Data represent mean ± SEM. Statistical significance was determined by unpaired *t* test (B, E) and between two means using post hoc Holm-Sidak multiple comparison tests (G). **P<0.01, ***P<0.005.

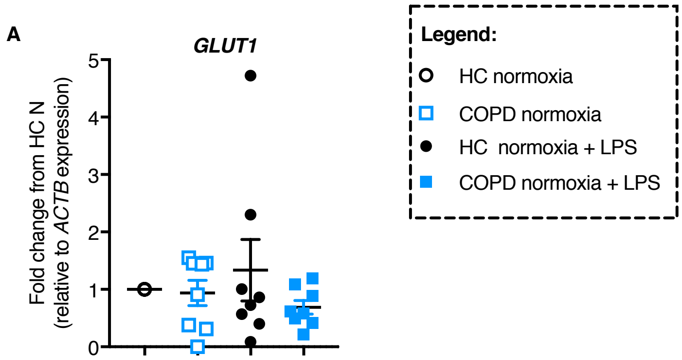

Figure S6. *GLUT1* transcript expression in HC and COPD peripheral blood neutrophils. Related to figure 5

GLUT1 transcript expression in HC and COPD neutrophils cultured for 6 h in the presence and absence of LPS, normalized to β-actin expression. Data are expressed as individual data points with mean +/- SEM, n=8.3

Age (years)	58.7 (40-76)
Gender (M:F)	10:14
$FFEV_1$(L) (forced expiratory volume in 1 second)	1.6 (0.7-2.5)
FEV1% (forced expiratory volume in 1 second %)	60 (33-98)
COPD GOLD stage I:II:III:IV	5:12:4:1
Smoking status (Ex:Current)	11:13
Pack years	40.2 (20-80)

Table S1. COPD patient demographics. Related to figure 5
